# Comprehensive Profiling Reveals Distinct Microenvironment and Metabolism Characterization of Lung Adenocarcinoma

**DOI:** 10.3389/fgene.2021.619821

**Published:** 2021-05-28

**Authors:** Chang Li, Chen Tian, Yangyang Liu, Jinyan Liang, Yulan Zeng, Qifan Yang, Yuting Liu, Di Wu, Jingjing Wu, Juanjuan Wang, Kai Zhang, Feifei Gu, Yue Hu, Li Liu

**Affiliations:** Cancer Center, Union Hospital, Tongji Medical College, Huazhong University of Science and Technology, Wuhan, China

**Keywords:** molecular subtype, tumor microenvironment, prognosis, immune escape, lung adenocarcinoma, bioinformactics analysis, metabolism

## Abstract

Lung adenocarcinoma has entered into an era of immunotherapy with the development of immune checkpoint inhibitors (ICIs). The identification of immune subtype is crucial to prolonging survival in patients. The tumor microenvironment (TME) and metabolism have a profound impact on prognosis and therapy. The majority of previous studies focused on only one aspect, while both of them are essential to the understanding of tumorigenesis and development. We hypothesized that lung adenocarcinoma can be stratified into immune subgroups with alterations in the TME infiltration. We aimed to explore the “TME-Metabolism-Risk” patterns in each subtypes and the mechanism behind. Glycolysis and cholesterol were selected for the analysis of metabolic states based on the first half of the study. Bioinformatic analysis was performed to investigate the transcriptomic and clinical data integrated by three lung adenocarcinoma cohorts (GSE30219, GSE31210, GSE37745, *N* = 415). The results were validated in an independent cohort (GSE50081, *N* = 127). In total, 415 lung adenocarcinoma samples were integrated and analyzed. Four major immune subtypes were indentified using bioinformatic analysis. Subtype NC1, characterized by a high level of glycolysis, with extremely low microenvironment cell infiltration. Subtype NC2, characterized by the “Silence” and “Cholesterol biosynthesis Predominant” metabolic states, with a middle degree infiltration of microenvironment cell. Subtype NC3, characterized by the lack of “Cholesterol biosynthesis Predominant” metabolic state, with abundant microenvironment cell infiltration. Subtype NC4, characterized by “Mixed” metabolic state, with a relatively low microenvironment cell infiltration. Least absolute shrinkage and selection operator (LASSO) regression and multivariate analyses were performed to calculate the risk of each sample, and we attempted to find out the potential immune escape mechanism in different subtypes. The result revealed that the lack of immune cells infiltration might contribute to the immune escape in subtypes NC1 and NC4. NC3 was characterized by the high expression of immune checkpoint molecules and fibroblasts. NC2 had defects in activation of innate immune cells. There existed an obviously survival advantage in subtype NC2. Gene set enrichment analysis (GSEA) and Gene Ontology analysis indicated that the PI3K-AKT-mTOR, TGF-β, MYC-related pathways might be correlated with this phenomenon. In addition, some differentially expressed genes (DEGs) were indentified in subtype NC3, which might be potential targets for survival phenotype transformation.

## Introduction

Lung adenocarcinoma is one of the most frequent cause of cancer-related death with a low 5-year survival rate (Hirsch et al., 2017; Bray et al., 2018). High rate of invasion and metastasis are major problems in lung adenocarcinoma. Great advancements in the treatment of lung adenocarcinoma have been achieved in the past few decades. Now immunotherapy, especially immune checkpoint blockage treatment, has become an emerging paradigm in cancer treatment, which is thought one of the most promising modalities for cancer treatment (Herbst et al., 2018; Saito et al., 2018). However, the efficacy of ICIs (immune checkpoint inhibitors) varies widely between individuals (Zaretsky et al., 2016), and the survival rate of lung adenocarcinoma remains low. In addition, metabolic reprogramming is an important characteristic of lung adenocarcinoma, which can result in tumor immune evasion and immunosuppression (Li X. et al., 2019). Therefore, more research into immune escape mechanism and potential therapeutic targets is required to improve the therapeutic effect of immunotherapy and to expand the benefits to a larger population (Mascaux et al., 2019).

The development of next generation sequencing has deepened our understanding of cancers, and researchers have developed various algorithms to estimate the abundance of specific cell types (Newman et al., 2015; Becht et al., 2016; Petitprez et al., 2018; Xiao et al., 2019). The public databases, such as gene expression omnibus (GEO) and ImmPort, have made it possible to explore the microenvironment and metabolism of tumor (Bhattacharya et al., 2014; Clough and Barrett, 2016). However, previous studies have focused on only tumor microenvironment (TME) or tumor cell metabolism, which might lead to an incomplete understanding of lung adenocarcinoma (Darvin et al., 2018; Sanchez-Vega et al., 2018; Chow et al., 2019; Havel et al., 2019; Zeng et al., 2019), as there’s a profound relationship between microenvironment and metabolism.

In this study, the survival, microenvironment and metabolic state of patients with lung adenocarcinoma were analyzed by data mining. We classified 415 lung adenocarcinoma samples into four clusters with distinct TME-Metabolism state, which might have differences in immune escape mechanisms and prognosis. In addition, we found several molecules which could be potential biomarkers for the treatment of lung adenocarcinoma.

## Materials and Methods

### Lung Adenocarcinoma Data Sets and Preprocessing

A total of four datasets were selected. Three of them (GSE30219, GSE31210, and GSE37745) were integrated as a training group, including 417 patients. Meanwhile, 127 patients from GSE50081 was treated as a validation group. All microarray data are from the Affymetrix platform (GPL570, Affymetrix Human Genome U133 Plus 2.0 Array). The raw data were downloaded from the Gene Expression Omnibus^1^, gene set was downloaded from the ImmPort database, which covered 1,242 genes.

The procedure used for data preprocessing was as follows. (1) Extracting expression data of lung adenocarcinoma patients; (2) removing samples with no clinical information; (3) robust-multi-array average (RMA) algorithm (Gautier et al., 2004) was performed for data background adjustment and quantile normalization in Affy R package; (4) surrogate variable analysis (SVA) algorithm (Leek et al., 2012) were used to eliminate the batch effects; (5) preserving the expression profiles of immune-related genes as immune-genes expression profile. Finally, we arrived at a training group of 415 patients and a validation group of 127 patients, as well as their immune-genes expression profiles, respectively.

Detail information about sample preprocessing is shown in the Supplementary Materials and Methods (Supplementary Figure 1).

### Consensus Clustering to Identify LUAD Immune Subtypes

The ConsensusClusterPlus algorithm (Wilkerson and Hayes, 2010) was performed (“kmeans” function in R, reps = 1,000, pItems = 0.8, pFeature = 1, distance = Euclidean) to determine molecular subtypes based on immune-gene expression profiles. The optimal “K” was determined by CDF (cumulative distribution function) curves. The top 100 upregulated genes in each immune subtype were identified using Limma package. False discovery rate (FDR) was calculated using the Benjamini-Hochberg method, and results with FDR < 0.05 were considered statistically significant. PCA (principal component analysis) is a statistical method in feature extraction and data analysis. The top upregulated genes were subjected to PCA to confirm the stability of the consensus clustering. For heatmap (heatmap, R package), we utilized the consensus clustering result to sort the samples to check immune-genes expression in each subtypes.

### Prognostic Analysis of LUAD Immune Subtypes and Construction of the Prognostic Prediction Model

The Kaplan–Meier method was utilized to plot survival curves, and log-rank test were used to compare OS rates of all immune subtypes. Differentially expressed genes (DEGs) were identified using Limma package, and survival-related genes were identified by univariate Cox regression analysis. LASSO (least absolute shrinkage and selection operator) regression analysis is a common method to solve the collinearity problems (Hu et al., 2017) via the implementation of a penalty proportional to their size, and it preserves the advantages of subset shrinkage. The data was subsampled 1000 times and immune genes which repeated > 900 times were chosen (glmnet in R). Among all survival-related genes, key genes were selected by LASSO regression analysis and subjected to multivariate Cox regression analysis to construct immune-related prognostic prediction model, using the regression coefficients derived from multivariate Cox regression analysis. The Kaplan–Meier method and log-rank test were utilized to compare the OS rates in different risk groups. Receiver operating characteristic curve (survival ROC in R) analysis was further utilized to assess the predictive ability of the prognostic prediction model. The riskscore model is described as follows:

$$\text{Riskscore}=\sum_{i=1}^{n}\beta \,i*X\,i$$

where *Xi* represents the expression value of the gene and β*i* represents the coefficients of the each gene derived from multivariate Cox regression analysis.

### Comparison of Enriched Hallmark Pathways and Gene Ontology Analysis

To identify hallmark pathways enriched among cluster 2 and other clusters, we performed a gene set enrichment (Subramanian et al., 2005) analysis (GSEA in R). All gene sets were downloaded from mSigDB (Liberzon et al., 2011), and GSEA was performed in R using hypergeometric tests. The threshold was set at corrected *P* < 0.05. Subsequently, we compared each pathway enriched among these clusters. The Database for Annotation, Visualization and Intergrated Discovery (DAVID) was utilized to perform functional analysis to determine the biological function of the survival-related immune genes. Significant biological processes were integrated and visualized using the Goplot (ggalluvial in R). The cut-off criteria was based on the threshold of *P* < 0.05.

### Estimation of TME Cell Abundance

Single sample gene set enrichment (Hanzelmann et al., 2013) analysis (GSVA in R) is a method to calculated the abundance of each cell subset in each sample. Two gene signatures, MCPcounter and CIBERSORT (Newman et al., 2015; Becht et al., 2016; Petitprez et al., 2018), were used to construct our gene sets. CIBERSORT is a popular approach to calculate the 22 immune cells (LM22) abundance of tissues based on their gene expression profiles, especially for microarray data from the Affymetrix platform. MCPcounter contains signatures of endothelial and fibroblasts cells, which were of significance to LUAD microenvironment. In all, we utilized ssGSEA to calculate the abundance of 24 LUAD microenvironment cells. The microenvironment score, which contains immune score and stromal score, was calculated using ESTIMATE R package (Yoshihara et al., 2013).

### Estimation of Metabolic State of Each Subtype

To estimate the metabolic state of each subtype, two gene sets “Cholesterol biosynthesis” and “Glycolysis” from mSigDB (Liberzon et al., 2011) were utilized to calculate the metabolic state of glycolysis and cholesterol in each sample. GSVA was used to estimate the level of the two metabolic gene sets. After *z*-score transformation, we stratified the samples into four groups: silence (glycolysis ≤ 0, cholesterol biosynthesis ≤ 0), marked as A. Cholesterol biosynthesis predominant (glycolysis ≤ 0, cholesterol biosynthesis > 0), marked as B. Glycolysis predominant (glycolysis > 0, cholesterol biosynthesis ≤ 0), marked as C. Mixed type (glycolysis > 0, cholesterol biosynthesis > 0), marked as D.

### Immunohistochemistry

Two lung adenocarcinoma tissues and paired paracarcinoma were used for immunohistochemical staining (IHC). In brief, paraffin-embedded tissue sections were deparaffinized, rehydrated, and pretreated for epitope retrieval. After blocked with 5% goat serum for an hour, the sections were incubated with appropriate primary antibodies overnight at 4 degrees. The primary antibodies used were from Abcam/Santa Cruz/Servicebio/Invitrogen: ADIPOR1 (1:500, ab126611, Abcam), ARRB1 (1:200, ab32099, Abcam), S100A12 (1:20, sc-101347, Santa Cruz), CD1b (1:200, Abcam, ab173576), HAMP (1:50, sc-101347, Santa Cruz), HMOX1 (1:1000, GB11845, Servicebio), KL (1:200, ab181373, Abcam), S100A7 (1:20, sc-52948, Santa Cruz), S100A2 (1:2000, GB111077, Servicebio), VEGFA (1:20, sc-7269, Santa Cruz), VIPR1 (1:50, Invitrogen, PA3-113), and TUBB3 (1:50, sc-80016, Santa Cruz). Following incubation with an HRP-conjugated secondary antibody (1:300, K8002, Dako), the stained sections were reacted with 3,3’-diaminobenzidine and counterstained with hematoxylin.

### Statistical Analysis

The normality of data was tested by Shapiro-Wilk normality test. Ordered categorical variables were analyzed by Wilcoxon test and Kruskal–Wallis test. Student’s *t* test was utilized to compare continuous variables. We utilized Fisher’s exact test or chi-square test to analyze the relationship between clinical variables and immune subtypes. Correlation analysis was performed by Spearman correlation. Survival analysis was performed using Kaplan–Meier curves and log-rank test. All statistical tests were two sided, and *P* < 0.05 was regarded to be statistically significant. The FDR correction was performed to decrease false positive rates in multiple tests. All statistical analyses were performed with R software (version 3.5.3)^2^.

## Results

### Identification of Lung Adenocarcinoma Immune Subtypes Based on Immune-Related Genes

**FIGURE 1:**
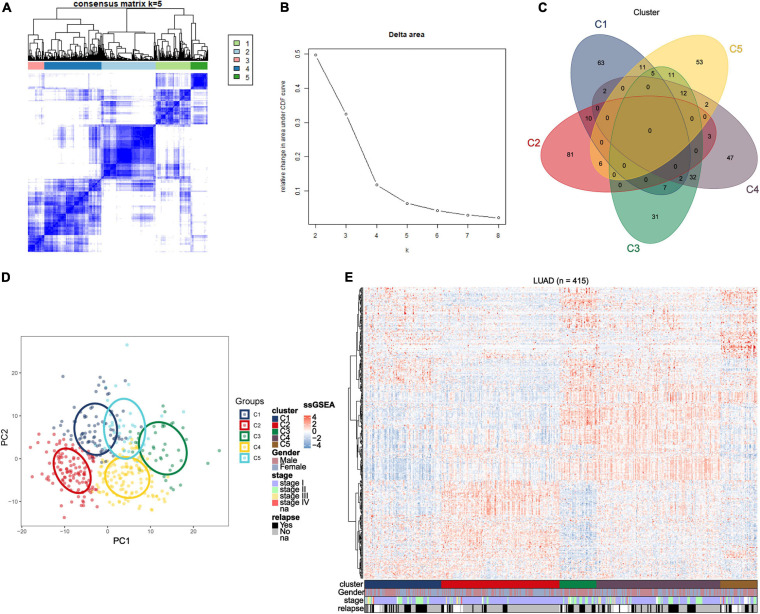
Identification of immune-related subtypes of lung adenocarcinoma in training group. **(A)** Heatmap of top 100 immune-related genes upregulated in each subtypes. Red and blue indicate relatively high or low expression value. **(B)** The results of the unsupervised consensus clustering. The consensus matrix for the optimal cluster number *k* = 5. **(C)** Relative change in area under the cumulative distribution function curves based on different subtype number (*k* = 2, 3, 4, 5, 6, 7, and 8). **(D)** Principal component analysis of expression profiles of the top upregulated immune-related genes in each subtype. Each immune subtype is represented with a unique color. **(E)** Venn diagram of top 100 upregulated immune-related genes in each subtype, with numbers represent the number of genes overlapping in a specific pair of subtypes.

We obtained raw data of three cohorts (GSE30219, GSE31210, and GSE37745, *N* = 415) from the GEO database. After standardization and adjustments, a gene expression profiles of 1242 immune-related genes was utilized to identify the lung adenocarcinoma subtypes (Supplementary Table 1). Unsupervised consensus clustering (K-means) was utilized to investigate different clusters. In detail, the procedure was performed with 100% gene resampling and 80% items resampling 1,000 times, and distance metric was calculated using Euclidean distances (Figure 1A). Complete clustering results are shown in Supplementary Figure 2. The optimal clustering result was obtained when *k* = 5 based on the “Delta area” plot, as the five cluster is the largest number induced the least change in the area under the CDF curves (Figure 1B). Thus, we separated the 415 LUAD samples into five subtypes based on the immune-related genes expression profiles, and the subtypes were labeled as C1 (*N* = 81), C2 (*N* = 125), C3 (*N* = 39), C4 (*N* = 131), and C5 (*N* = 39).

The top 100 upregulated immune genes in each immune subtype were calculated. Importantly, there were 46 top genes overlapping between C3 and C4, while only relatively fewer genes overlapped in other pairs of clusters (Figure 1C), revealing a moderate level of similarity between subtypes C3 and C4. Principal component analysis was performed using the top upregulated immune genes. The PCA result indicated that these genes were divided into five types (Figure 1D), and the unsupervised consensus clustering result was further validated by PCA analysis. Moreover, a heatmap of top genes in each immune subtypes was plotted to investigate the gene expression pattern of each subtypes (Figure 1E). As shown in the figure, in terms of immune-related genes, each subtype showed different expression patterns. These results prompted that the subtypes may have diverse immune microenvironment.

### Correlation Between the Clinical Characteristics and Subtypes

**FIGURE 2:**
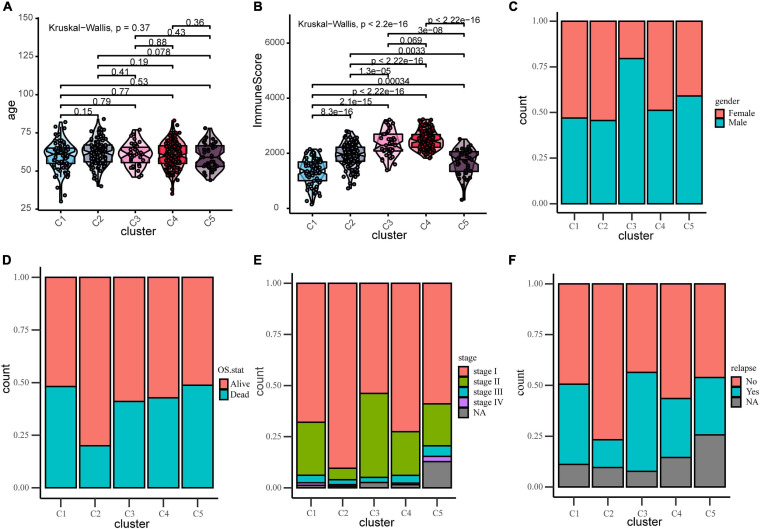
Distribution analyses of five immune subtypes based on some clinical characteristics or ESTIMATE immune-scores. **(A)** Age distribution in the five LUAD immune subtypes. **(B)** Immune scores distribution in the five LUAD immune subtypes. **(C)** Gender proportion in the five subtypes. **(D)** Survival status proportion in the five subtypes. **(E)** Stage proportion in the five subtypes. **(F)** Relapse status proportion in the five subtypes.

To find the correlation between the clinical features and different immune subtypes, the clinical information of the training group was collated, and several clinical factors were analyzed (including age, gender, survival status, relapse, and stage). There was no significant difference in age distribution (Figure 2A) among five immune subtypes (Kruskal–Wallis test, *P* > 0.05). The distribution of gender (Figure 2C) among subtypes were not significantly different except subtype C3, which could have been ascribed to the relatively small sample size of C3. Results of Chi-square test were shown in Supplementary Table 5. These results adjusted the effects of confounding factors age and gender, which were not main target of our study. Of the survival status, the proportion of “alive” and “dead” (Figure 2D) differed among five subtypes (Chi-square test *P* < 0.05). The proportion of “alive” status was significantly higher in C2, and the proportion of “dead” status was relatively higher in C1 and C5. Regarding tumor stage, as shown in the figure, most patients were at stage I and stage II in each subtypes. The proportion of stage I (Figure 2E) in C2 and the proportion of stage II in C3 were relatively higher (Chi-square test *P* < 0.05). Next, we investigated the proportion of relapse in each subtypes (Figure 2F), and the result indicated that the proportion of “no relapse” was significantly higher in subtype C2 as compared to other subtypes (Chi-square test *P* < 0.05). In addition, the distribution of immune scores in each subtypes was calculated (ESTIMATE function in R), and the result (Figure 2B) revealed that there was a significant difference among five subtypes (Kruskal–Wallis test, *P* < 0.001). The average immune score in subtypes C3 and C4 were relatively higher while the average score was relatively low in C1 and C5. The immune score in subtype C2 was at middle level.

### Prognostic Significance of Immune Subtypes and Construction of Riskscore Model

**FIGURE 3:**
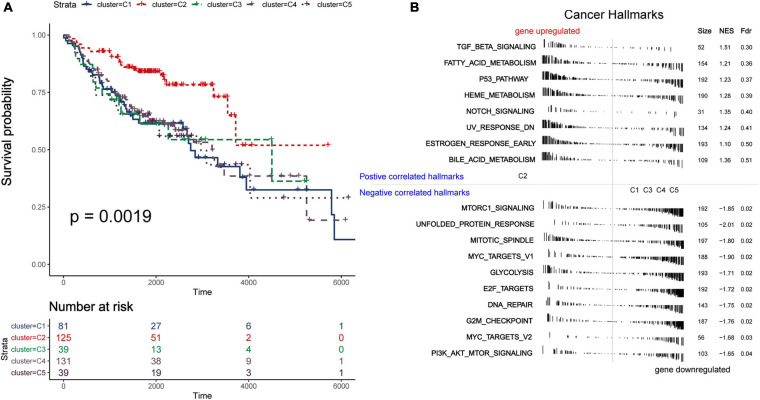
Survival and function analyses of immune subtypes. **(A)** Gene set enrichment analysis of immune subtypes in training group. Significant pathways in subtype C2 and the other subtypes. **(B)** Kaplan–Meier plot of overall survival of different subtypes. Log-rank test is used for statistical significance. **(C)** Kaplan–Meier plot of overall survival of different clusters (integrate C3 and C4 as a whole).

Due to the invasive and metastatic potential of lung adenocarcinoma, the 5-year survival rates for patients with LUAD remains low. Considering the different immune-related genes expression patterns in five subtypes, which may play an important role in tumor prognosis, we utilized survival analysis to investigate the relevence between clinical outcome and the five subtypes (Figure 3A). The Kaplan–Meier curves revealed a distinct survival difference among the immune subtypes (OS, log-rank test *P* = 0.0019). The LUAD patients in subtype C2 had significantly better overall survival than the other subtypes. As a matter of fact, the other subtypes (C1, C3, C4, and C5) had the similar clinical outcomes, which were worse than in subtypes C2. It should be noted that the subtype C3 and C4 had the highest immune score but didn’t show the advantages of overall survival, and the mechanism behind this phenomenon was worth exploring. In general, C2 was the subtype with a better prognosis. The differentially expressed immune-related genes in subtype C2 were identified using limma package (FDR < 0.05, absolute log2-fold change > 1). We utilized DAVID database (The Database for Annotation, Visualization and Integrated Discovery) to perform functional enrichment analysis for the DEGs. Important biological process and functions were integrated and visualized in a chord diagram (Supplementary Figure 4). According to the results, the DEGs in subtype C2 were mainly enriched in cell chemotaxis, regulation of cell proliferation and apoptotic (“regulation of T cell proliferation,” “regulation of apoptotic process”), immune response and inflammatory response. To further explore the mechanism behind the survival difference between subtype C2 and the other subtypes, GSEA analysis based on the transcriptome profile was performed (Figure 3B). The results indicated that the gene signatures of “TGF-beta signaling,” “Notch signaling,” and “Bile acid metabolism” were enriched in subtype C2, while other gene signatures, such as “Glycolysis” and “PI3K-AKT-MTOR signaling,” were enriched in the other subtypes. The detailed results were shown in Supplementary Table 8. It can be concluded that these biological process and hallmark pathways play a significant role in the survival of patients with lung adenocarcinoma. We direct compared the survival outcome for Glycolysis high vs. low patients and PIK3-AKT-MTOR high vs. low patients in the training and validation cohorts (Supplementary Figure 5). The results revealed that patients in high glycolysis group had worse survival outcome as compared to low Glycolysis group both in the training and validation cohorts (Log-rank test, *P* < 0.001, *P* < 0.001, respectively, Supplementary Figures 5A,C). Patients in high PIK3-AKT-MTOR signaling group had worse survival outcome in the training cohort (log-rank test, *P* < 0.001, Supplementary Figure 5B), but the results didn’t reach statistical significance in the validation cohort (log-rank test, *P* = 0.27).

**FIGURE 4:**
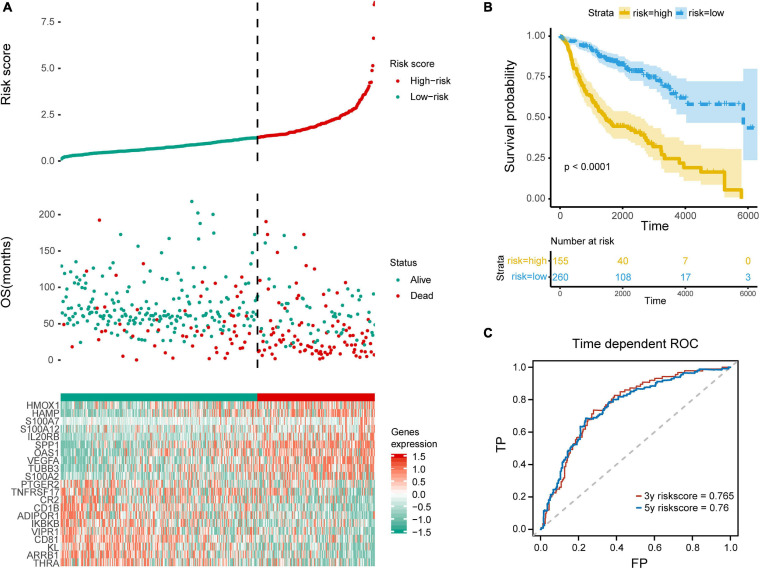
Evaluation of the prediction model. **(A)** Top row, the low and high risk score group for the immune-related mRNA signature in training group; middle row, the survival status and overall survival time of patients in training group; bottom row, heatmap showing the expression level of the genes involved in the risk score model. **(B)** Kaplan–Meier plot of overall survival of patients with lung adenocarcinoma, with blue for low risk and yellow for high risk. **(C)** ROC analysis shows the risk score model AUC = 0.765 at 3-year and 0.760 at 5-year, respectively.

In order to distinguish the survival risk among different immune subtypes, we have constructed a prognostic prediction model. First, 205 immune-related genes significantly correlated with overall survival were identified using univariate Cox regression analysis. LASSO regression analysis (1,000 times) based on the L1-penalized estimation was performed to obtain genes with the greatest prognostic values. 21 genes (ADIPOR1, ARRB1, CD1B, CD81, CR2, HAMP, HMOX1, IKBKB, KL, S100A2, S100A7, SPP1, THRA, TNFRSF17, TUBB3, VEGFA, VIPR1, IL20RB, OAS1, PTGER2, and S100A12) that appeared > 900 times out of 1,000 repetitions were selected to be analyzed (Table 1). Multivariate Cox regression analysis was performed to determine the coefficient of each gene. Finally, the prognostic prediction model was constructed (riskscore = −0.311 × normalized expression level of ADIPOR1 – 0.242 × normalized expression level of ARRB1 – 0.168 × normalized expression level of CD1B – 0.001 × normalized expression level of CD81 – 0.087 × normalized expression level of CR2 + 0.187 × normalized expression level of HAMP + 0.052 × normalized expression level of HMOX1 – 0.205 × normalized expression level of IKBKB + 0.011 × normalized expression level of IL20RB + 0.016 × normalized expression level of KL + 0.124 × normalized expression level of OAS1 – 0.210 × normalized expression level of PTGER2 + 0.066 × normalized expression level of S100A12 + 0.009 × normalized expression level of S100A2 + 0.102 × normalized expression level of S100A7 + 0.068 × normalized expression level of SPP1 – 0.421 × normalized expression level of THRA – 0.162 × normalized expression level of TNFRSF17 + 0.047 × normalized expression level of TUBB3 + 0.219 × normalized expression level of VEGFA – 0.067 × normalized expression level of VIPR1). In addition, we used the genotype tissue expression (GTEx) dataset, together with The Cancer Genome Atlas (TCGA) data, to compare the mRNA expression between tumor and normal tissues (Supplementary Figure 12). The risk score of each sample in training group was calculated, and we categorized the patients into high or low risk groups based on best cut-off calculated by X-tile software (Table 2) (Long et al., 2019). Genes involved in the model and the corresponding HR were shown in Supplementary Figure 6D. The distributions of the risk scores, OS, survival status, and corresponding mRNA expression profiles of the 415 patients in the training group are shown in Figure 4A. The protective mRNA (VIPR1, TNFRSF17, THRA, PTGER2, KL, IKBKB, CR2, CD81, CD1B, ARRB1, and ADIPOR1) tended to be more highly expressed in the low-risk group, while the remaining mRNA (VEGFA, TUBB3, SPP1, S100A7, S100A2, S100A12, OAS1, IL20RB, HMOX1, and HAMP) were more highly expressed in the high-risk group. Moreover, the high-risk group had more death than the low-risk group. The Kaplan–Meier plot (Figure 4B) indicated that the patients in low-risk group had a significant survival advantage compared to the high-risk group (log-rank test *P* < 0.001). Time-dependent ROC analysis (Sing et al., 2005) was performed to show the predictive potential of the prognostic prediction model. The area under the ROC curve (AUC) of the prognostic model for overall survival was 0.765 at 3 years and 0.760 at 5 years, respectively (Figure 4C). These results indicated that the model had a good predictive ability.

**TABLE 1:** mRNAs involved in the prognosis prediction model of LUAD.

**Gene symbol**	**Ensemble ID**	**Coefficient**	**HR**
ADIPOR1	ENSG00000159346	−0.311	0.568
ARRB1	ENSG00000137486	−0.242	0.680
CD1B	ENSG00000158485	−0.168	0.707
CD81	ENSG00000110651	−0.001	0.529
CR2	ENSG00000117322	−0.087	0.708
HAMP	ENSG00000105697	0.187	1.399
HMOX1	ENSG00000100292	0.052	1.351
IKBKB	ENSG00000104365	−0.205	0.700
IL20RB	ENSG00000174564	0.011	1.163
KL	ENSG00000133116	0.016	0.774
OAS1	ENSG00000089127	0.124	1.208
PTGER2	ENSG00000125384	−0.210	0.807
S100A12	ENSG00000163221	0.066	1.248
S100A2	ENSG00000196754	0.009	1.167
S100A7	ENSG00000143556	0.102	1.138
SPP1	ENSG00000118785	0.068	1.187
THRA	ENSG00000126351	−0.421	0.452
TNFRSF17	ENSG00000048462	−0.162	0.878
TUBB3	ENSG00000258947	0.047	1.875
VEGFA	ENSG00000112715	0.219	1.293
VIPR1	ENSG00000114812	−0.067	0.697

**TABLE 2:** Univariate and multivariate analyses of age, gender, stage, and riskscore with overall survival in training cohort.

	**Univariate analysis**		**Multivariate analysis**	
	**HR (95% CI)**	***P* value**	**HR (95% CI)**	***P* value**
**Training group (*N* = 405)**				
Age	1.042 (1.021–1.064)	<0.001	1.043 (1.021–1.066)	<0.001
Gender	1.205 (0.867–1.674)	0.268		
Stage	1.807 (1.466–2.227)	<0.001	1.548 (1.237–1.938)	<0.001
Risk score	1.418 (1.287–1.563)	<0.001	1.369 (1.233–1.520)	<0.001

### Microenvironment Landscape of Immune Subtypes and Potential Immune Escape Mechanism

**FIGURE 5:**
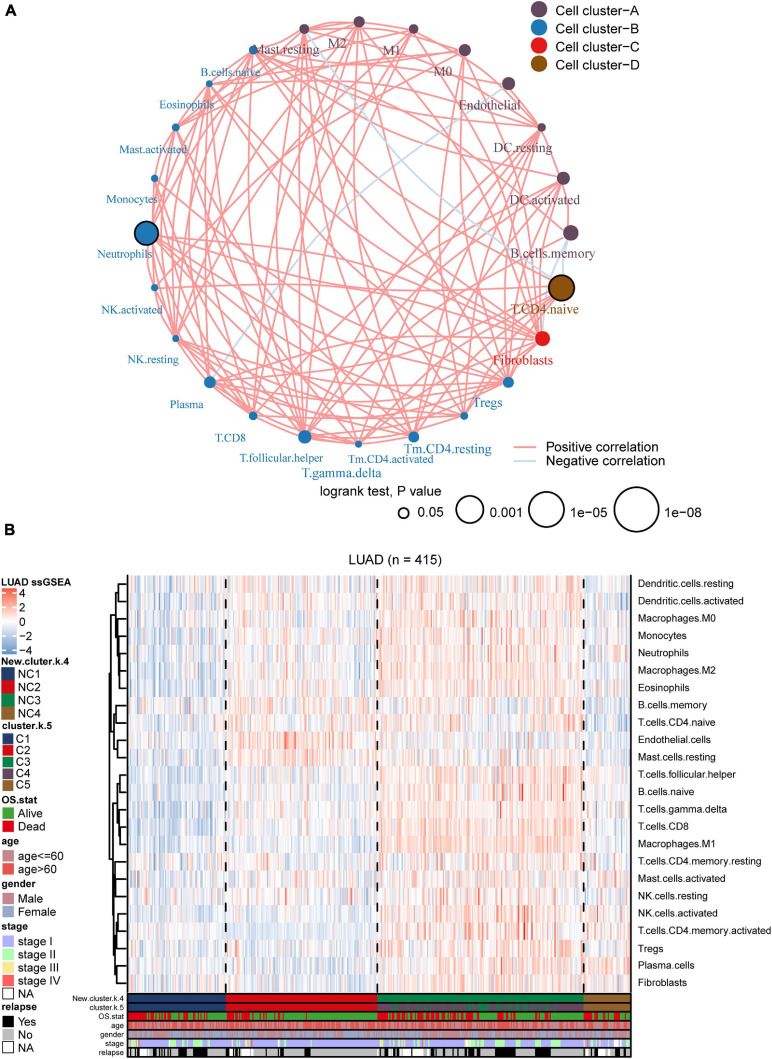
Landscape of the subtypes. **(A)** Interaction plot of the tumor microenvironment cell types. The color of each cell represents the cluster of each cell type, calculation used Spearman correlation analysis. The size of each circle indicates the survival impact of each cell type. The connecting lines represent the correlation between TME cells, with red for positive correlation and blue for negative correlation. **(B)** The heatmap indicates the TME cells distribution among different subtypes (*k* = 5 or 4), with red for high expression and blue for low expression, and it is annotated by age, stage, gender, survival status, and relapse status.

We utilized ssGSEA to calculate the abundance of 24 TME cells (including endothelial cells, fibroblasts, and 22 immune cells). The comprehensive landscapes of LUAD microenvironment cell interactions and their effects on the OS of patients were integrated into a network diagram (Figure 5A). The specific results were shown in the Supplementary Table 2. Three types of TME cell (Memory B cells, Neutrophils and Activated memory CD4 T cells) showed significant difference in overall survival (*P* < 0.05). As shown in the network, there existed strong connection among different TME cells. The heatmap was drawn to depict the distribution of 24 TME cells among different immune subtypes (Figure 5B). Notably, the distributions of 24 TME cells in subtypes C3 and C4 were very similar. As mentioned earlier, many immune-related genes were identified overlapped between subtypes C3 and C4, and the two subtypes showed similarly high immune-scores. Thus, subtypes C3 and C4 were integrated as a new group for further analysis. In addition, we directly compared the results of consensus clustering when *k* = 4 and 5 (Supplementary Table 7), and it indicated that C3 and C4 had strong consistency. To facilitate making the distinction among different LUAD immune subtypes, the former C1, C2, C5 was labeled as NC1, NC2, and NC4, respectively. The subtypes C3 and C4 were integrated and labeled as NC3. The heatmap revealed that the microenvironment cells infiltration had clear different patterns among the 4 subtypes (NC1, NC2, NC3, and NC4). NC1 was characterized by extremely low microenvironment cells infiltration, while NC3 was characterized by high degree microenvironment cells infiltration. NC2 and NC4 had middle degree and low degree immune infiltration, respectively. The proportions of 22 immune cells were calculated and shown (Supplementary Figure 7) in boxplot (one-way ANOVA test). The majority of immune cells had different proportion among NC immune subtypes, such as Macrophages M2, Treg cells, which might play a significant role in the immune escape mechanism.

**FIGURE 6:**
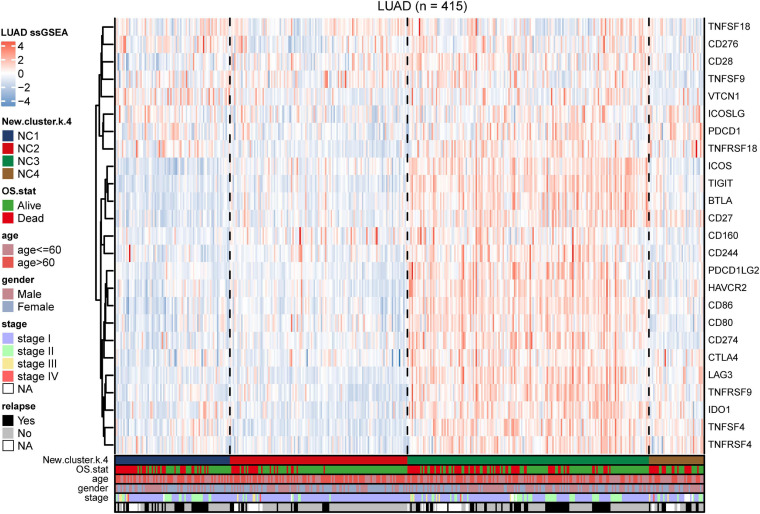
Immune checkpoint relevant molecules in four subtypes. The heatmap reveals the expression of 25 immune checkpoint relevant molecules of four subtypes, with red for high expression and blue for low expression. The heatmap is annotated by stage, age, gender, survival status, and relapse status.

To further investigate the intrinsic immune escape mechanisms of different immune subtypes, 25 immune checkpoint relevant molecules expression among the four NC subtypes were analyzed. The heatmap was drawn (Figure 6) to show the different expression patterns of immune checkpoint molecules in four subtypes, and it indicated that the expression of checkpoint molecules were obviously higher in subtype NC3 than the other subtypes. The detail information was shown in the boxplot (Supplementary Figure 8). The average expression of 19 molecules (CTLA, CD160, CD244, CD27, CD274, CD28, CD80, CD86, CTLA4, HAVCR2, ICOS, IDO1, LAG3, PDCD1LG2, TIGIT, TNFRSF18, TNFRSF4, TNFRSF9, and TNFSF4) in subtype NC3 was relatively higher compared to other subtypes (ANOVA test), while CD276, VTCN1 had higher expressions in subtype NC1, and PDCD1 had a higher expression in subtype NC4, respectively.

### Identification of Metabolic State of the Immune Subtypes

**FIGURE 7:**
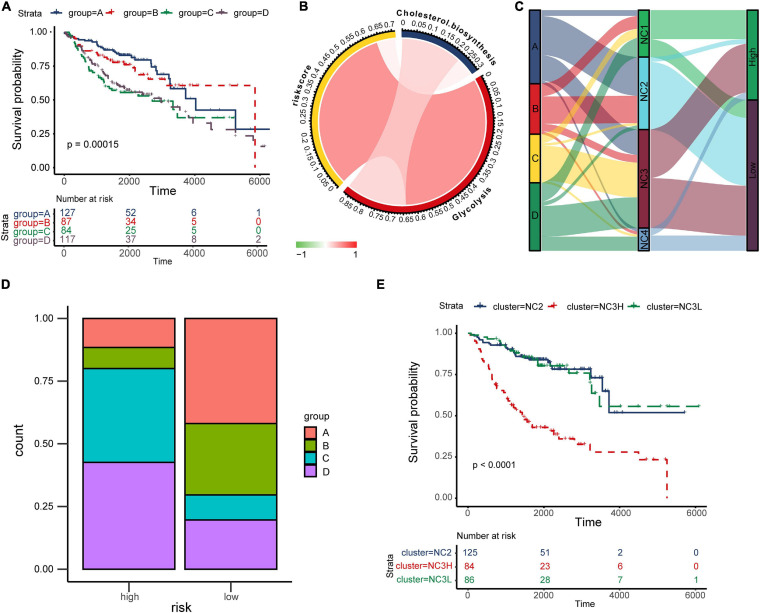
The “TME-Metabolism-Risk” patterns in training group and the specificity of subtype NC3. **(A)** Kaplan–Meier plot of different metabolic group. The statistical difference was compared through log-rank test. **(B)** The correlation among Cholesterol biosynthesis, glycolysis and riskscore. **(C)** Sankey plot showing the correlation among metabolic states, immune subtypes and the survival risk. **(D)** The metabolic states proportion in high/low risk groups. **(E)** Kaplan–Meier plot of subtypes NC2, NC3L, and NC3H. The statistical difference was compared through the log-rank test.

We utilized the 14 tumor metabolism relevant gene sets to estimated the metabolic state in the training group (including adenosine metabolic process, arginine biosynthetic,

cyclooxygenase, fatty acid biosynthetic, lactate metabolic process, one carbon metabolic process, oxidative phosphorylation, cholesterol biosynthesis, glutamate and glutamine metabolism, glycogen synthesis, glycolysis, fatty acid beta oxidation, pentose phosphate pathway, and tryptophan catabolism, Supplementary Figure 9). The results indicated that there were significant differences in metabolic states among different subtypes. K–M plots (Supplementary Figure 10) indicated that several metabolism pathways (adenosine metabolic process, glutamate and glutamine metabolism, glycogen synthesis, and glycolysis) were related to overall survival. According to study of Karasinska et al. (2019), we chose “glycolysis” and “cholesterol biosynthesis” to classified metabolic state. GSVA was performed to calculate the enrichment degree of both “glycolysis” and “cholesterol biosynthesis” metabolic pathways in each sample (*N* = 415). The metabolic states were stratified into four types and were labeled as A, B, C, D as mentioned before (A for Silence, B for Cholesterol biosynthesis Predominant, C for Glycolysis Predominant, and D for Mixed type). Survival analysis was performed to investigate the relevence between metabolic states and immune subtypes (NC1-4). Kaplan–Meier plot (Figure 7A) indicated that there existed an obviously overall survival difference among four metabolic groups (log-rank test *P* < 0.01). Survival benefits were observed in group A and B (A vs. C, log-rank test *P* < 0.01, A vs. D, log-rank test *P* < 0.01, B vs. C log-rank test *P* < 0.05, B vs. D log-rank test *P* < 0.01). However, the survival differences had no statistical significance in other pairs of groups (A vs. B, C vs. D, log-rank test *P* > 0.05). The chord diagram (Figure 7B) revealed an obviously positive correlation between riskscore and Glycolysis (Spearman rho = 0.64, *P* < 0.05) and a relatively weak correlation between Glycolysis and Cholesterol biosynthesis (Spearman rho = 0.23, *P* < 0.05).

In the TME, metabolic adaptation allow cancer cells to survive. There existed a deep connection between the TME and tumor metabolism. The Sankey plot (Figure 7C) was drawn to reveal the relationship between the three characteristics (tumor metabolic states, immune subtypes, and risk groups). The main types of metabolism in different subtypes were shown clearly in the plot (NC1: C, D; NC2: A, B; NC3: A, C, D; NC4: D). In most cases, NC2 led to the low survival risk while NC1 and NC4 contributed to the high survival risk. Comparing the proportion of metabolic states (Figure 7D) in different risk groups in subtype NC3, we found the proportions of metabolic states C, D in the high-risk group were obviously higher than in the Low-risk group. Interestingly, the subtype NC3 which had a high degree microenvironment cells infiltration, obtained obviously differentiation in the survival risk. This phenomenon led us to further explore the survival characteristics of NC3. Kaplan–Meier plot indicated that the NC3L (low-risk patients in subtype NC3) had the apparently survival advantage, and the similar advantage was also observed in subtype NC2 (Figure 7E). DEGs between NC3L and NC3H (High-risk patients in subtype NC3) were calculated using the limma R package. These molecules (Supplementary Table 3) might play an important role in the high-low risk transformation in NC3, and the mechanism behind this might ascribed to the different metabolic states of NC3.

### External Validation

**FIGURE 8:**
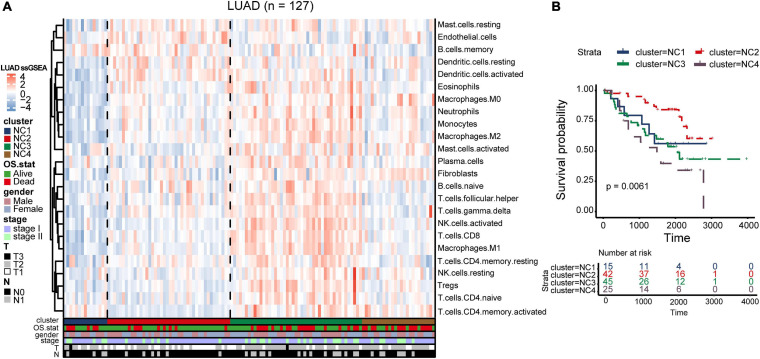
TME Validation in cohort GSE50081. **(A)** The expression heatmap of TME cell types in the four subtypes of validation group. Red represents high expression and blue represents low expression. **(B)** Kaplan–Meier plot of overall survival of four immune subtypes. The statistical difference was compared through the log-rank test.

**FIGURE 9:**
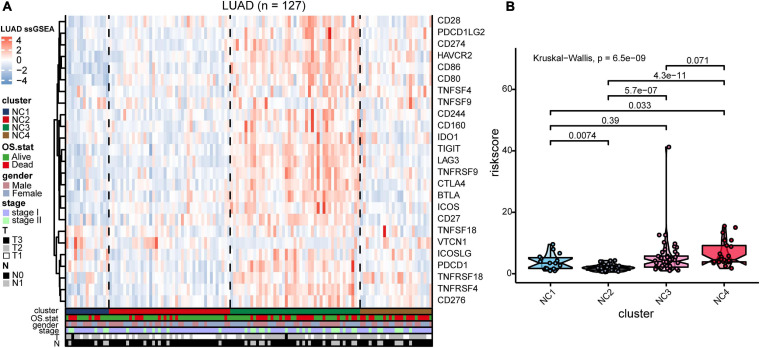
IC Validation in cohort GSE50081. **(A)** The expression heatmap of the same 25 immune checkpoint relevant molecules in the four subtypes. **(B)** Riskscore distribution in the four subtypes. The statistical difference was compared through the Kruskal–Wallis test.

To validate our findings, we utilized a cohort of patients of lung adenocarcinoma (GSE50081, Supplementary Tables 4, 6) as our validation group (*N* = 127). We independently applied the unsupervised clustering algorithm on the validation dataset using the ConsensusClusterPlus (*k*-means function in R). The clustering results (Supplementary Figure 3) were very similar to the results identified in the training group. The heatmaps revealed the TME cells infiltration and the expression level of 25 immune checkpoint relevant molecules in validation group (Figures 8A, 9A). The subtype NC3 had a high degree of TME cells infiltration, and the expression level of immune checkpoint molecules were obviously higher compared to the other clusters (Figure 9A). NC1 was characterized by extremely low degree of immune infiltration, while NC2 and NC4 had middle and relatively low degree of infiltration, respectively. An apparently overall survival advantage was observed in subtype NC2 (Figure 8B).

The riskscore of each sample in validation group was calculated (Figure 9B), and the receiver operating curve was plotted based on the riskscore of each sample. As presented in the plot (Supplementary Figure 6C), AUC for overall survival was 0.755 at 3 years and 0.757 at 5 years, respectively. There was a significant survival difference in prognosis between the high-risk group and low-risk group (Supplementary Figure 6B, log-rank test *P* < 0.001). The similar “metabolic states-immune subtypes-risk groups” patterns were also found in the validation group as the Sankey plot indicated (Figure 8B and Supplementary Figure 11). The subtype NC3 with high degree microenvironment cells infiltration had an apparent differentiation of risk (Supplementary Figure 11C). On the whole, the reliability and stability of the results we obtained from the training group were verified in the validation group.

In addition, Wang et al. (2021) study constructed a platform, OncoVar, which identified several important drive genes in each cancer type. Immune-related genes were selected and survival analysis was conducted (Supplementary Table 9). The result revealed that ACVR1B was associated with poor prognosis in lung adenocarcinoma (HR = 1.713, *P* < 0.1), and it indicated that ACVR1B might have potential value in further research.

### Immunohistochemistry

**FIGURE 10:**
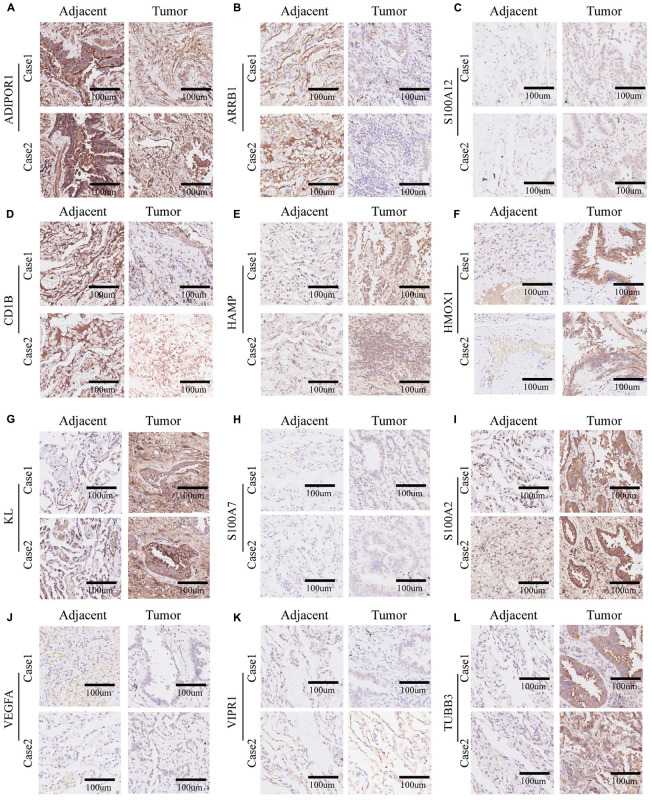
Immunohistochemistry of 12 selected genes expression in two lung adenocarcinoma cases. **(A–L)** Representative pictures of an IHC staining with paraffin-embedded tissue sections demonstrate the selected genes’ protein expression patterns (brown signal) in adjacent tissue (left panel) and matched malignant tumor tissue (right panel). The 12 selected genes were in order as follows: ADIPOR1, ARRB1, S100A12, CD1b, HAMP, HMOX1, KL, S100A7, S100A2, VEGFA, VIPR1, and TUBB3.

In order to verify the bioinformatics analysis results, we chose 12 genes from the 21 crucial immune genes(including ADIPOR1, ARRB1, CD1B, CD81, CR2, HAMP, HMOX1, IKBKB, KL, S100A2, S100A7, SPP1, THRA, TNFRSF17, TUBB3, VEGFA, VIPR1, IL20RB, OAS1, PTGER2, and S100A12) for further Immunohistochemical staining. Figures 10A–L show the staining patterns of the 12 chosen genes in adjacent tissue and matched malignant tumor tissue. The shade of brown indicates the level of specific proteins expression in the tissue. By and large, the protein expression level were consistent with the previous bioinformatics analysis: the protective genes (ADIPOR1, ARRB1, CD1B, KL, and VIPR1) were highly expressed in paracancer tissues compared with tumor tissues, while the remaining genes (HAMP, HMOX1, S100A12, S100A7, S100A2, TUBB3, and VEGFA) were more obvious in adenocarcinoma tissues.

## Discussion

Lung adenocarcinoma is characterized by the high rate of invasion and metastasis (Bray et al., 2018). The mortality and recurrence rate of lung adenocarcinoma could be reduced greatly with the early and optimal treatment. Great changes have taken place in the therapy of lung adenocarcinoma during the past few decades, and now, we are at the dawn of a new era of immunotherapy (Zaretsky et al., 2016; Sanchez-Vega et al., 2018; Chow et al., 2019; Zeng et al., 2019). A lot of efforts have been made to explore effective new targets for immunotherapy in cancers (Colombo et al., 2014). ICIs have begun to transform clinical treatment, and they are considered to be one of the most promising method in cancer therapy (Havel et al., 2019; Wei et al., 2018). However, the agents, such as pembrolizumab and nivolumab, only benefit a relatively limited population. Hence, it makes good sense to explore the mechanism behind the phenomenon, especially combined with the metabolic states. With the emergence of public databases, such as TCGA and GEO, data mining and analysis is becoming an important way to identify potential therapeutic target. The current study systematically analyzed the LUAD immune subtypes using the data extracted from the GEO cohorts.

The stage I and stage II patients contribute mostly to the proportion of the samples in our training group, thus the analysis results can represent the early stage patients quite well.

On the other hand, consensus clustering algorithm has been broadly utilized to discover significant clusters. According to the previous studies, the TME is segregated into three types based on the immune infiltration: immune desert, immune excluded and immune inflamed (Darvin et al., 2018). Our study revealed four important LUAD immune microenvironment subtypes and their clinical characteristics. Subtype NC1 was the so-called “extremely low immune infiltration” type, and it was characterized by the lack of “silence” metabolic state. Subtype NC2 had a middle degree of microenvironment cells infiltration, and the “silence” and “Cholesterol biosynthesis Predominant” metabolic modes contribute mostly to the proportion of metabolic states. Subtype NC3 was found to have a high degree of immune infiltration, and was characterized by the lack of “Cholesterol biosynthesis Predominant” metabolic state. The majority metabolic state in subtype NC4 was the “Mixed” type, and NC4 had a relatively low degree of microenvironment cells infiltration.

The extrinsic immune escape mechanism consists of four aspect: lack of immune cells, fibrosis, presence of immunoinhibitory cytokines and the immunoinhibitory cells, and the intrinsic immune escape consists of two major aspects: immune checkpoint molecules expression and tumor immunogenicity (Mascaux et al., 2019). The potential immune escape mechanisms were investigated, and we found the characteristics of the subtypes that might contribute to the immune escape. The lack of immune cells infiltration is the principal factor of immune escape mechanism in subtypes NC1 and NC4. Fibrosis and high expression of immune checkpoint molecules contribute to the immune escape mechanism in subtype NC3, while the subtype NC2 had defects in activation of innate immune cells.

In addition, we found an obviously survival advantage in subtype NC2. To figure out the mechanism behind this, GSEA and differential genes analysis were performed. The GSEA results revealed that the glycolysis and lipid metabolism might play an significant role in the survival of different subtypes (DeBerardinis et al., 2008; Levine and Puzio-Kuter, 2010). On the other hand, according to the study of Karasinska et al., glycolysis-cholesterol synthesis axis plays an important role in tumor development, and that was why we chose “glycolysis” and “cholesterol synthesis” metabolism pathways for further analysis. The results also revealed several hallmark pathways enriched in subtypes NC2 or the other subtypes. The PI3K-AKT-mTOR signaling pathway, which was enriched in the other subtypes, is a highly conserved. The activation of it enhances many tumor activities, including driving glycolysis, and the interruption of PI3K-AKT-mTOR pathway has been proved to change T cell metabolism (Li X. et al., 2019). The transforming growth factor-β is a key enforcer of tumor immune evasion and response, and it is generally considered related to immune suppression within TME (Batlle and Massague, 2019). The TGF-β can also be tumor suppressive through different approaches, such as a lethal EMT (David et al., 2016). This might account for the enrichment of TGF-β signaling in subtype NC2. The E2F-targets and MYC-targets pathways, which have been demonstrated to be associated with the relapse and cell proliferation in lung cancer, enriched in the groups with survival disadvantage.

In order to further elaborate the risk of patients with lung adenocarcinoma, we have constructed a prognostic prediction model based on immune-related genes. The prediction model consisted of 21 crucial immune genes, including ADIPOR1, ARRB1, CD1B, CD81, CR2, HAMP, HMOX1, IKBKB, KL, S100A2, S100A7, SPP1, THRA, TNFRSF17, TUBB3, VEGFA, VIPR1, IL20RB, OAS1, PTGER2, and S100A12. Previous study has demonstrated that the S100A family had a deep relationship with tumor development (Hatoum et al., 2017). Previous study has demonstrated that S100A2 had a deep relationship with the risk for colorectal cancer (Masuda et al., 2016). S100A7 plays an important roles in the development of estrogen receptor-positive breast carcinoma and non-small cell lung cancer (Lu et al., 2018; Mayama et al., 2018). S100A12 is closely related to vascular invasion by tumor cells, and causes excessive inflammation and vascular invasion, which lead to tumor recurrence and metastasis. It was found to play an important role in many human cancers, including breast cancer and papillary thyroid cancer (Gunaldi et al., 2015; Wang et al., 2020). SPP1 is an enzyme that dephosphorylates S1P (sphingosine 1-phosphate), which was found to mediate macrophage polarization (Le Stunff et al., 2002). Previous study revealed that it has the potential to serve as a prognostic biomark for lung adenocarcinoma (Zhang et al., 2018). IL20RB is the member of IL10 family, and it was considered crucial in autoimmune diseases and renal cell carcinoma (Yang et al., 2018; Cui et al., 2019). It has not been associated with LUAD prognosis before, which might serve as a potential target for LUAD.

The metabolic states of tumor and TME are inextricably linked. Various metabolic mechanisms could alter the behavior of TME cells (Li X. et al., 2019). In our study, we distinguished different metabolic states in four immune subtypes. Thus, we obtained the different “TME-Metabolism-Risk” patterns in our lung adenocarcinoma immune subtypes. It is worth noting that there existed a survival advantage in the “Cholesterol biosynthesis Predominant” group. Cholesterol are considered major risk factors in many diseases, including cancers (Baek et al., 2017). Previous studies have demonstrated that the cholesterol could induce CD8 positive T cell exhaustion in TME and facilitate breast cancer metastasis (Li J. et al., 2019). Our study revealed that, the cholesterol synthesis might be a crucial factor contributing to the suppressive of lung adenocarcinoma under the high level of glycolysis.

It should be noted that the subtype NC3 was characterized by high TME cells infiltration while the overall survival rate remained relatively low. Stratification analysis was utilized to investigate the survival differences among NC2, NC3L, and NC3H. The results revealed that the NC3H group had potential to transform into the better survival phenotype, and the DEGs might be the potential targets (Supplementary Table 3).

In general, we constructed a prognostic prediction model which provided good discrimination between high and low risk patients with lung adenocarcinoma. Then, our study indicated that the LUAD could be classified into four immune subtype with different characteristics, and this might facilitate the selection of treatment plans and the selection of appropriate patients for immunotherapy. For example, the subtype NC3 had a relatively high expression of immune checkpoint molecules, which might lead to the intrinsic immune escape, and patients categorized into this subtype may be particularly suitable to ICIs treatment while NC1 and NC4 with relatively low immune infiltration might not be appropriate for this treatment. Next, we identified the “TME – Metabolic state – Risk” patterns in each immune subtypes, and we found that the cholesterol synthesis was of particularity in lung adenocarcinoma. In conclusion, our study depicted the landscape of microenvironment and metabolism characterization of LUAD.

## Data Availability Statement

Publicly available datasets were analyzed in this study. This data can be found here: all data can be viewed in the GEO database (https://www.ncbi.nlm.nih.gov/geo/). The datasets analyzed for this study can be found here: GSE30219, GSE31210, GSE37745, and GSE50081.

## Author Contributions

LL, YyL, YH, JWu, and CL conceived and designed the study. CL, FG, JL, and DW acquired the data. YtL, CL, QY, CT, and KZ analyzed and interpreted the data. CL, YH, YtL, JWa, YZ, and KZ drafted the manuscript. All authors reviewed and revised this work, and gave their final approval of the submitted manuscript.

## Conflict of Interest

The authors declare that the research was conducted in the absence of any commercial or financial relationships that could be construed as a potential conflict of interest.

## Footnotes

1
https://www.ncbi.nlm.nih.gov/geo/

2
http://www.R-project.org
