# Corrigendum: Comprehensive profiling reveals distinct microenvironment and metabolism characterization of lung adenocarcinoma

**DOI:** 10.3389/fgene.2025.1585016

**Published:** 2025-04-03

**Authors:** Chang Li, Chen Tian, Yangyang Liu, Jinyan Liang, Yulan Zeng, Qifan Yang, Yuting Liu, Di Wu, Jingjing Wu, Juanjuan Wang, Kai Zhang, Feifei Gu, Yue Hu, Li Liu

**Affiliations:** Cancer Center, Union Hospital, Tongji Medical College, Huazhong University of Science and Technology, Wuhan, China

**Keywords:** molecular subtype, tumor microenvironment, prognosis, immune escape, lung adenocarcinoma, bioinformactics analysis, metabolism

In the published article, there was an error in Figure 10 as published. Specifically, the authors inadvertently assigned immunohistochemistry images from Case 1 patients to Case 2 patients in Figures 10D, K, and mistakenly used incorrect images in Figure10G. The corrected Figure 10 and its caption appear below.

**FIGURE 10:**
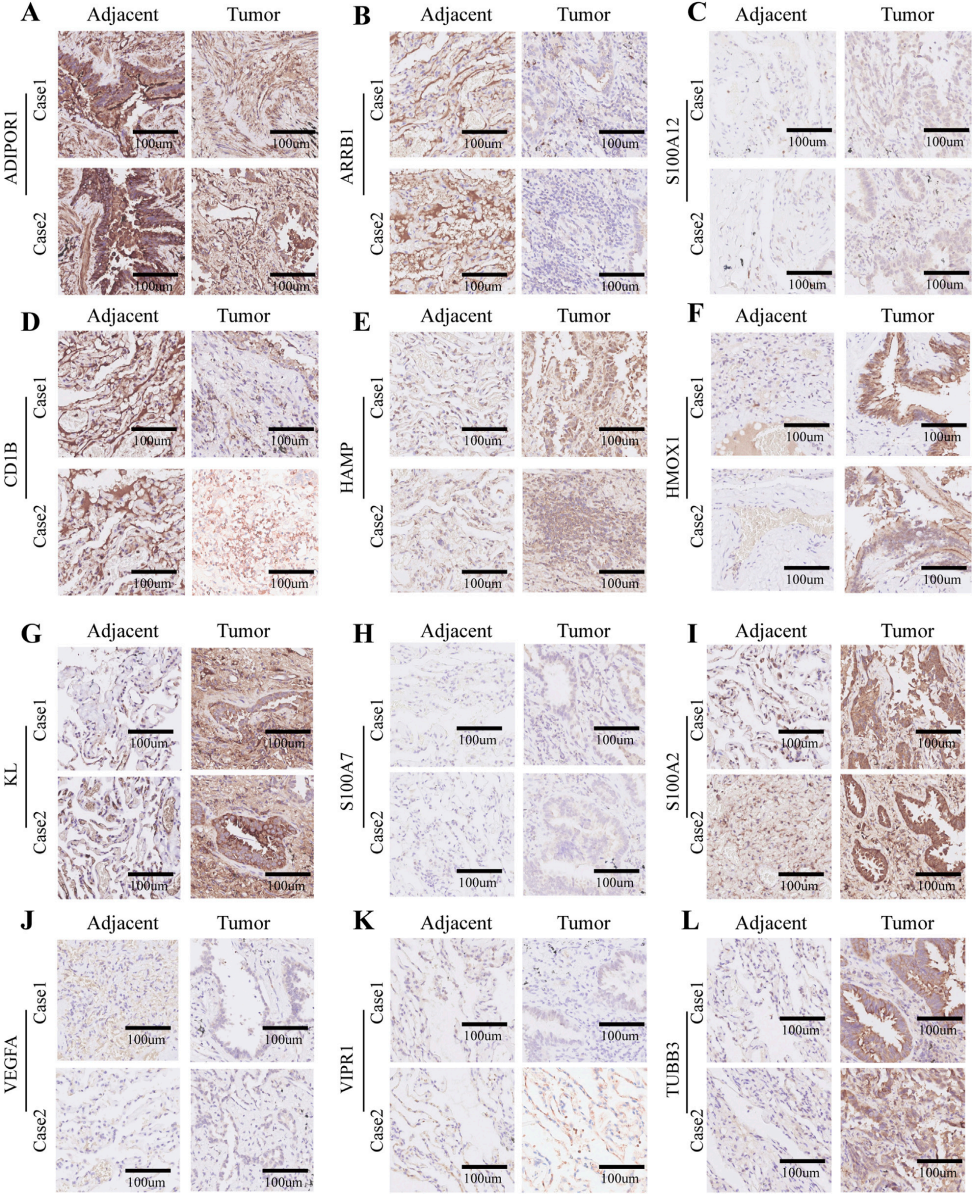
Immunohistochemistry of 12 selected genes expression in two lung adenocarcinoma cases. **(A–L)** Representative pictures of an IHC staining with paraffin-embedded tissue sections demonstrate the selected genes’ protein expression patterns (brown signal) in adjacent tissue (left panel) and matched malignant tumor tissue (right panel). The 12 selected genes were in order as follows: ADIPOR1, ARRB1, S100A12, CD1b, HAMP, HMOX1, KL, S100A7, S100A2, VEGFA, VIPR1, and TUBB3.

The authors apologize for these errors and state that this does not change the scientific conclusions of the article in any way. The original article has been updated.

