# Supplementary material for: Comprehensive Profiling Reveals Distinct Microenvironment and Metabolism Characterization of Lung Adenocarcinoma
Source: Front Genet. 2021 May 28;12:619821. doi: 10.3389/fgene.2021.619821 (PMC8193848; doi:10.3389/fgene.2021.619821)
Supplement: Supplementary file 1 [file Data_Sheet_1.PDF]

Figure S1

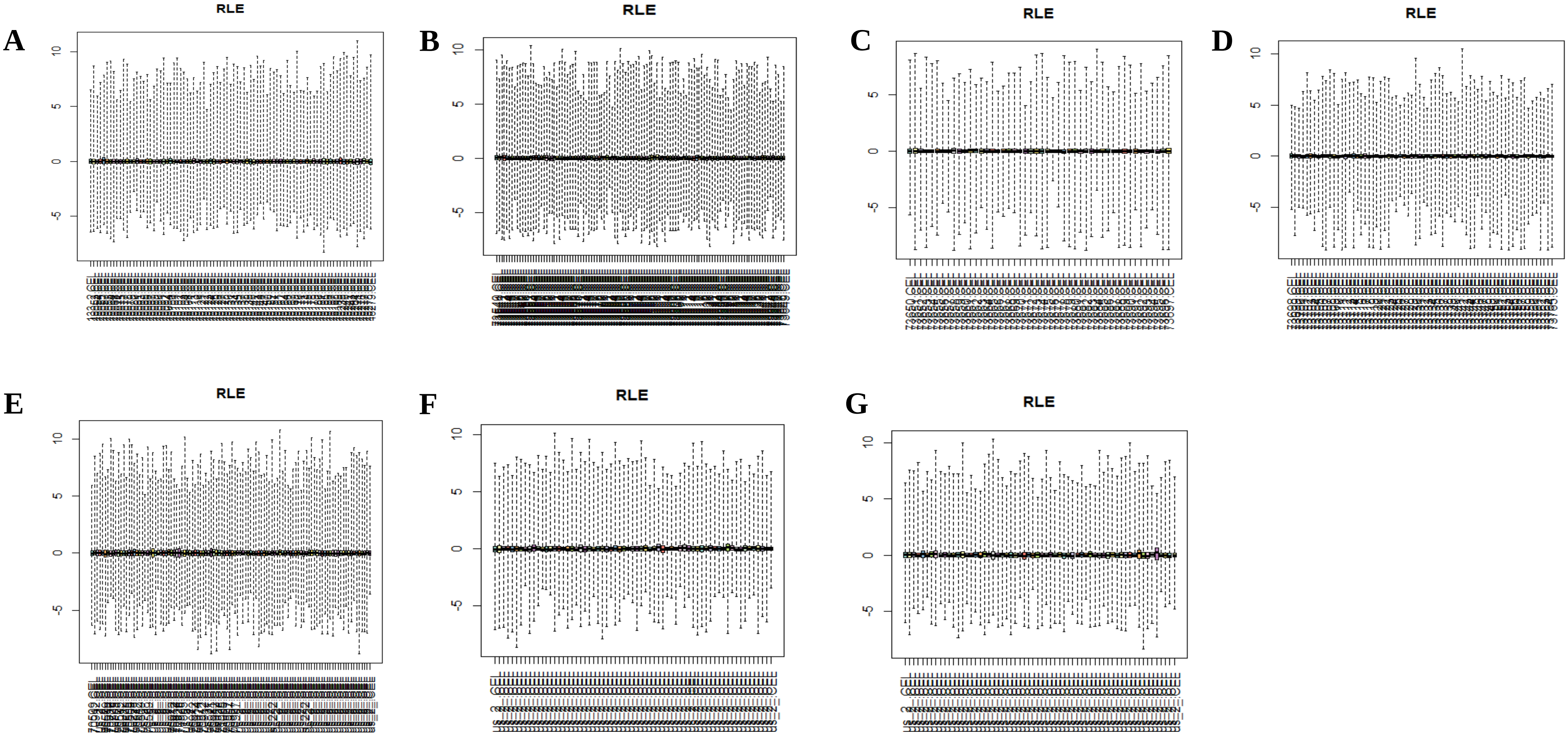

Fig S1. Diagram of data pre-processing. Relative log expression of raw data of four cohorts in our study. (A) represents GSE30219. (B), (C) and (D) represent GSE31210. (E) represent GSE37745. (F) and (G) represent GSE50081. No outlier was detected.

Figure S2

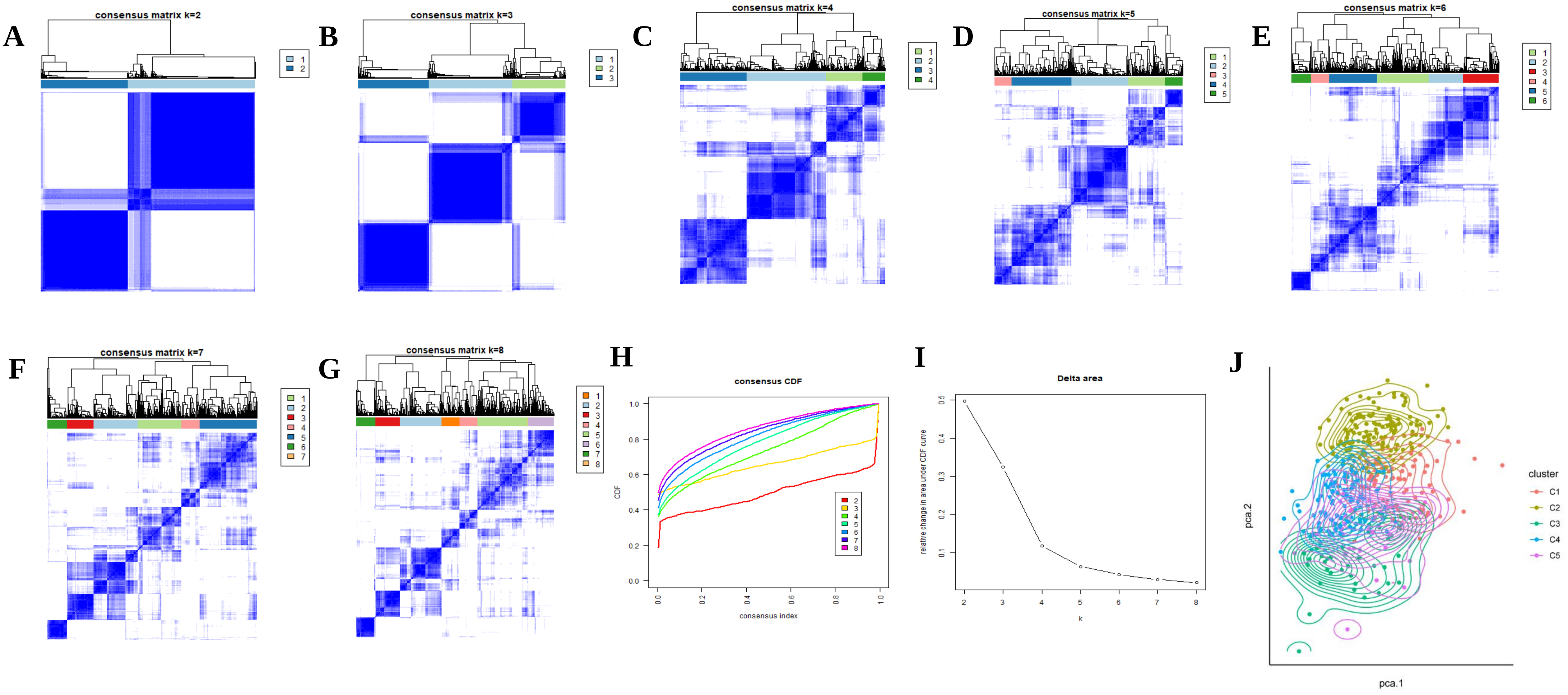

Fig S2. The results of consensus clustering in training group. (A)-(G) show the different consensus matrices based on the varying k (k=2,3,4,5,6,7,8). (H) Cumulative distribution function (CDF) curves of consensus scores based on different subtypes number. (I) Delta area curve indicates the relative change in area under CDF curves. K=4 or K=5 both seem to be the optimal clustering number. In order to avoid deviation maximumly, we first chose k=5 as the optimal number in our study. (J). Density contour of PCA of the top upregulated immune-related genes in each subtypes.

Figure S3

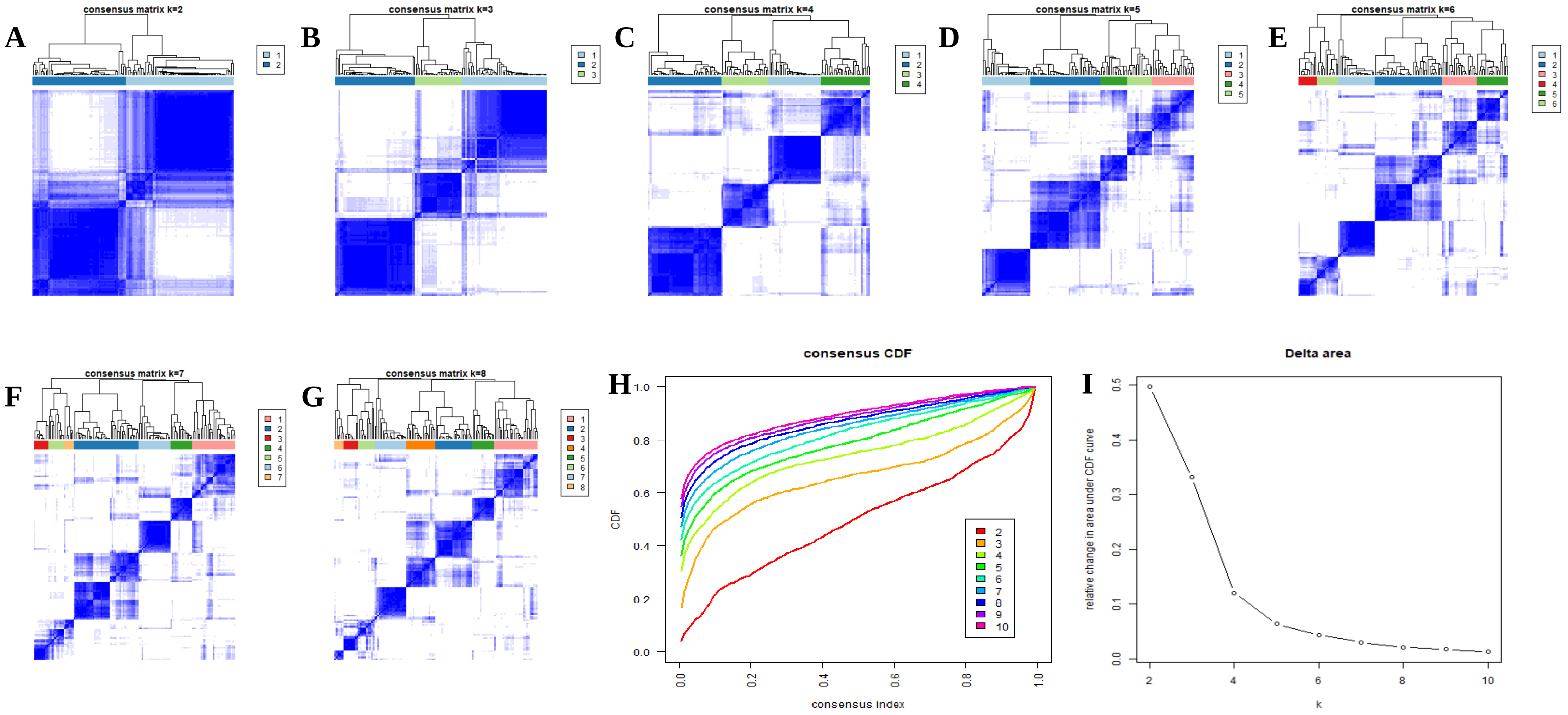

Fig S3. The results of consensus clustering in validation group. (A)-(G) show the different consensus matrices based on the varying k (k=2,3,4,5,6,7,8). (H) Cumulative distribution function (CDF) curves of consensus scores based on different subtypes number. (I) Delta area curve indicates the relative change in area under CDF curves. The similar results is obtained in the training group.

Figure S4

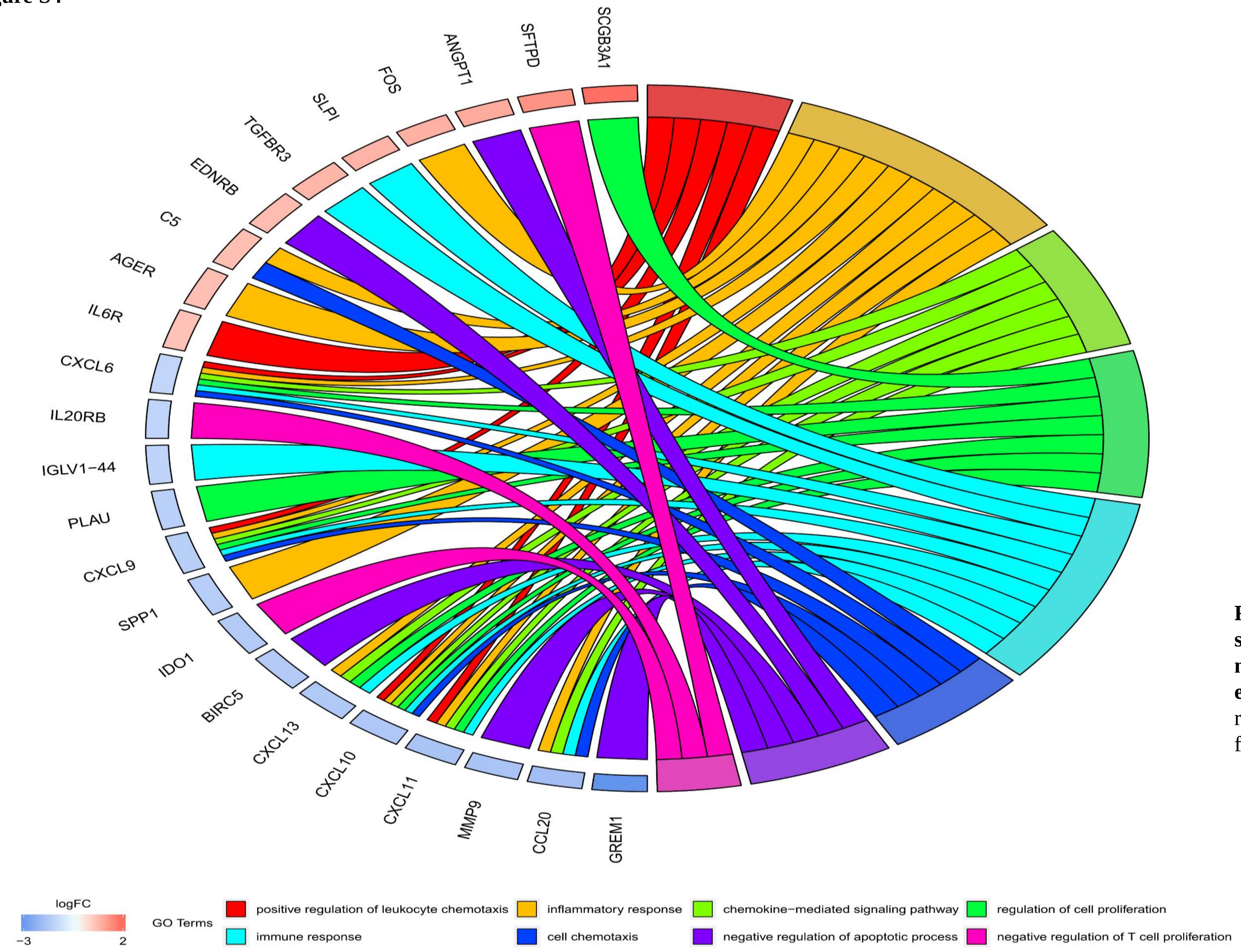

Figure S4. The chord diagram of significant biological processes and molecular function of differentially expressed genes in C2. Each chord represents a connection between genes and function (or processes).

Figure S5

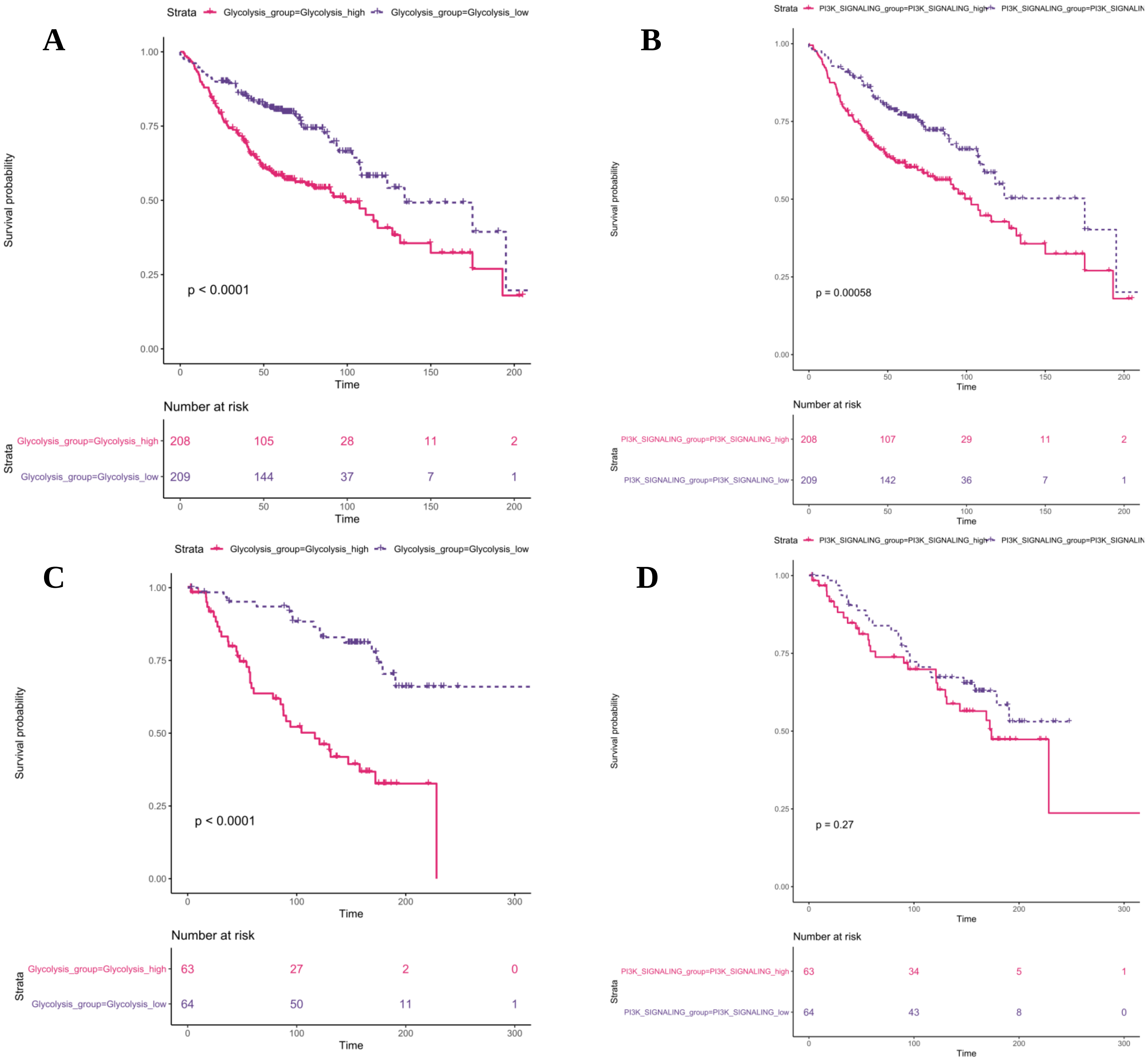

Figure S5. Direct comparison of the survival outcome for Glycolysis high vs low patients and PIK3-AKT-MTOR high vs low patients in the training and validation cohorts. (A). Kaplan-Meier plot of high/low Glycolysis in the training cohort. (B). Kaplan-Meier plot of high/low PI3K-AKT-MTOR high/low in the training cohort. (C). Kaplan-Meier plot of high/low Glycolysis in the validation cohort. (D). Kaplan-Meier plot of high/low PI3K-AKT-MTOR high/low in the validation cohort. The enrichment score of the two pathway were calculated by ssGSEA. The high/low groups were separated based on the median ssGSEA score. “PI3K_signaling” in the plot represented “PIK3-AKT-MTOR-signaling”.

Figure S6

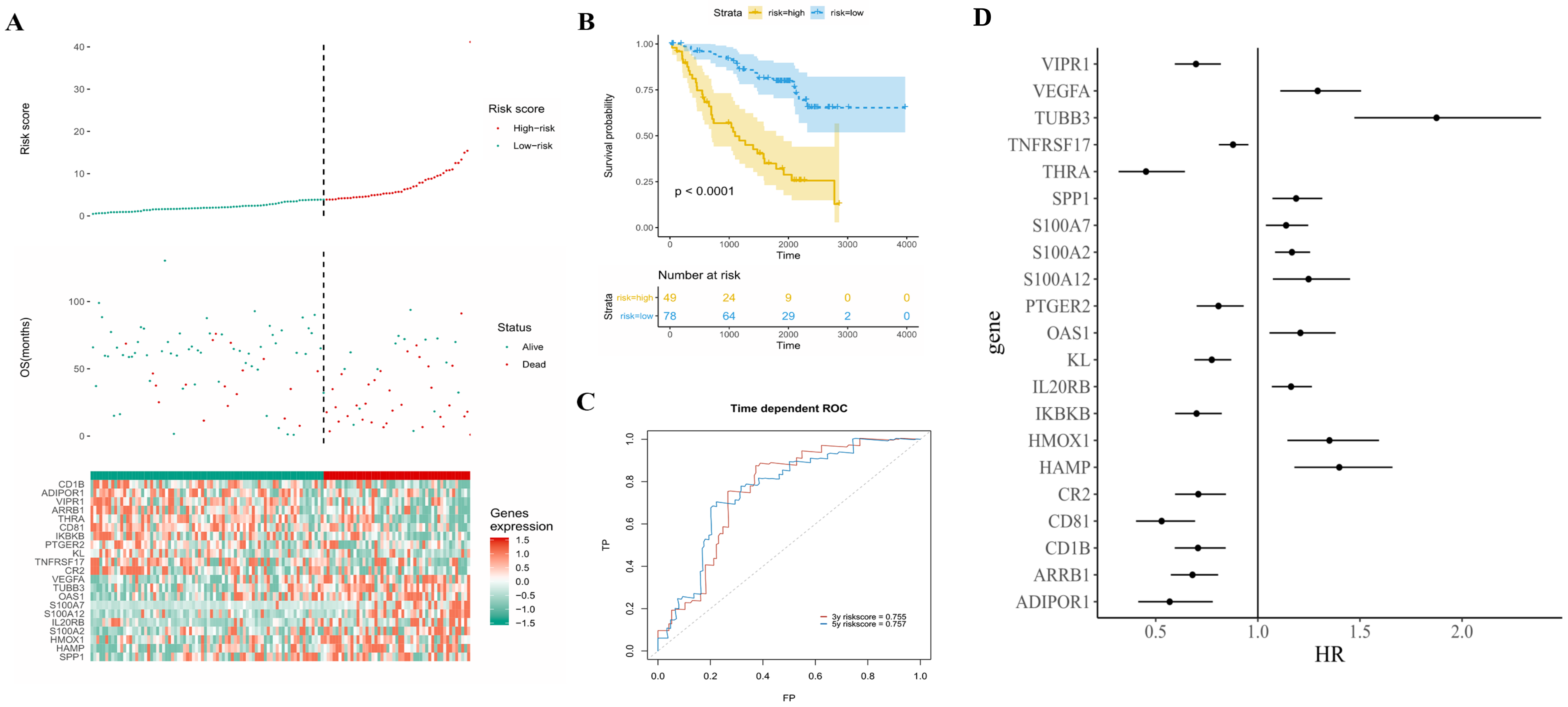

Figure S6. Analysis of risk score in the validation group. (A) Top row, the low and high risk score group for the immune-related mRNA signature in validation group; middle row, the survival status and overall survival time of patients in validation group; bottom row, heatmap showing the expression level of the genes involved in the risk score model. (B) Kaplan-Meier plot of overall survival of patients with lung adenocarcinoma, with blue for low risk and yellow for high risk. (C) ROC analysis shows the risk score model AUC = 0.755 at 3-year and 0.757 at 5-year, respectively. (D) Genes involved in the risk score model and the corresponding HR.

Figure S7

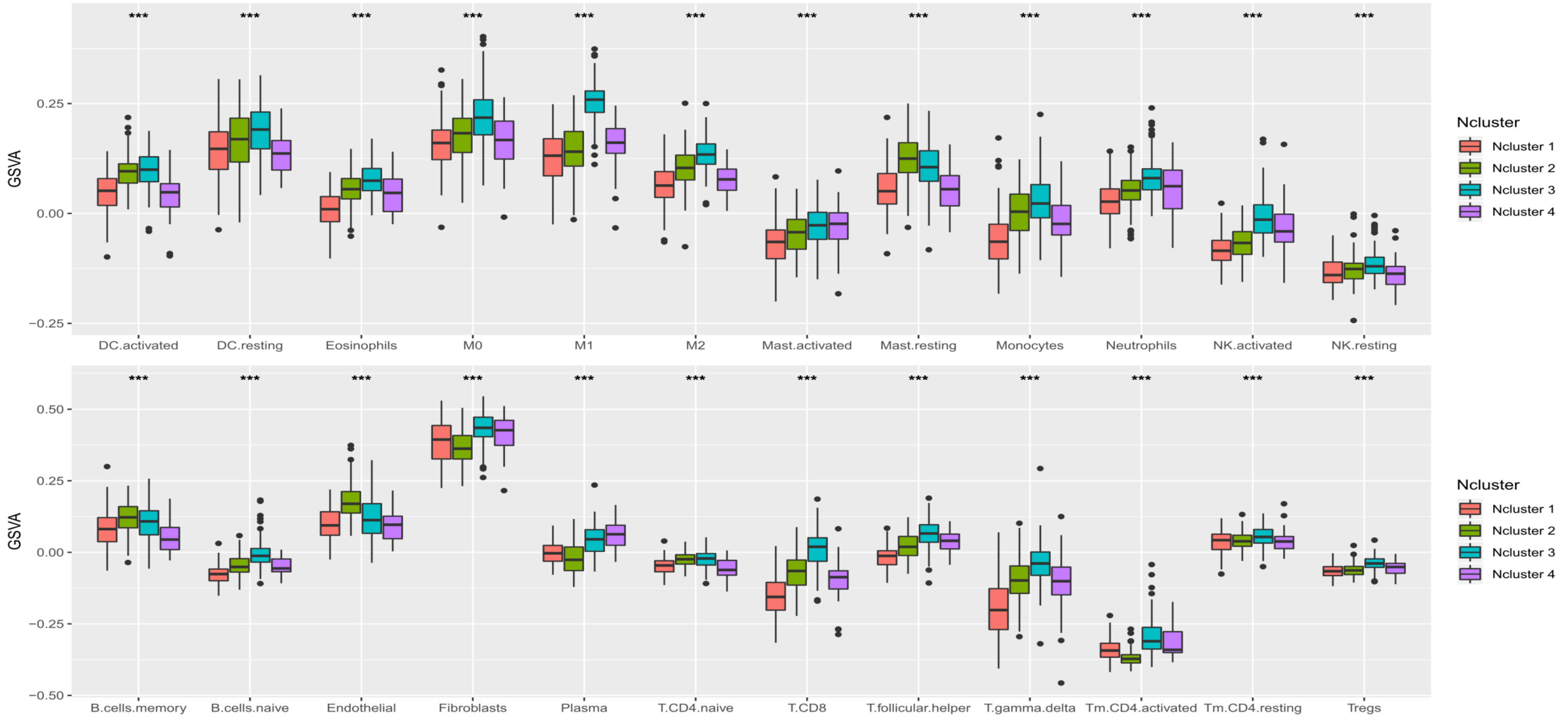

Figure S7. Boxplot of 24 TME cells across different immune subtypes in the training group. The boxplots show the medians and dispersions of the samples of different immune subtypes for each TME cells. P-values are the results of the one-way ANOVA for the different immune subtypes (NC1-NC4). *** $p<0.001$

Figure S8

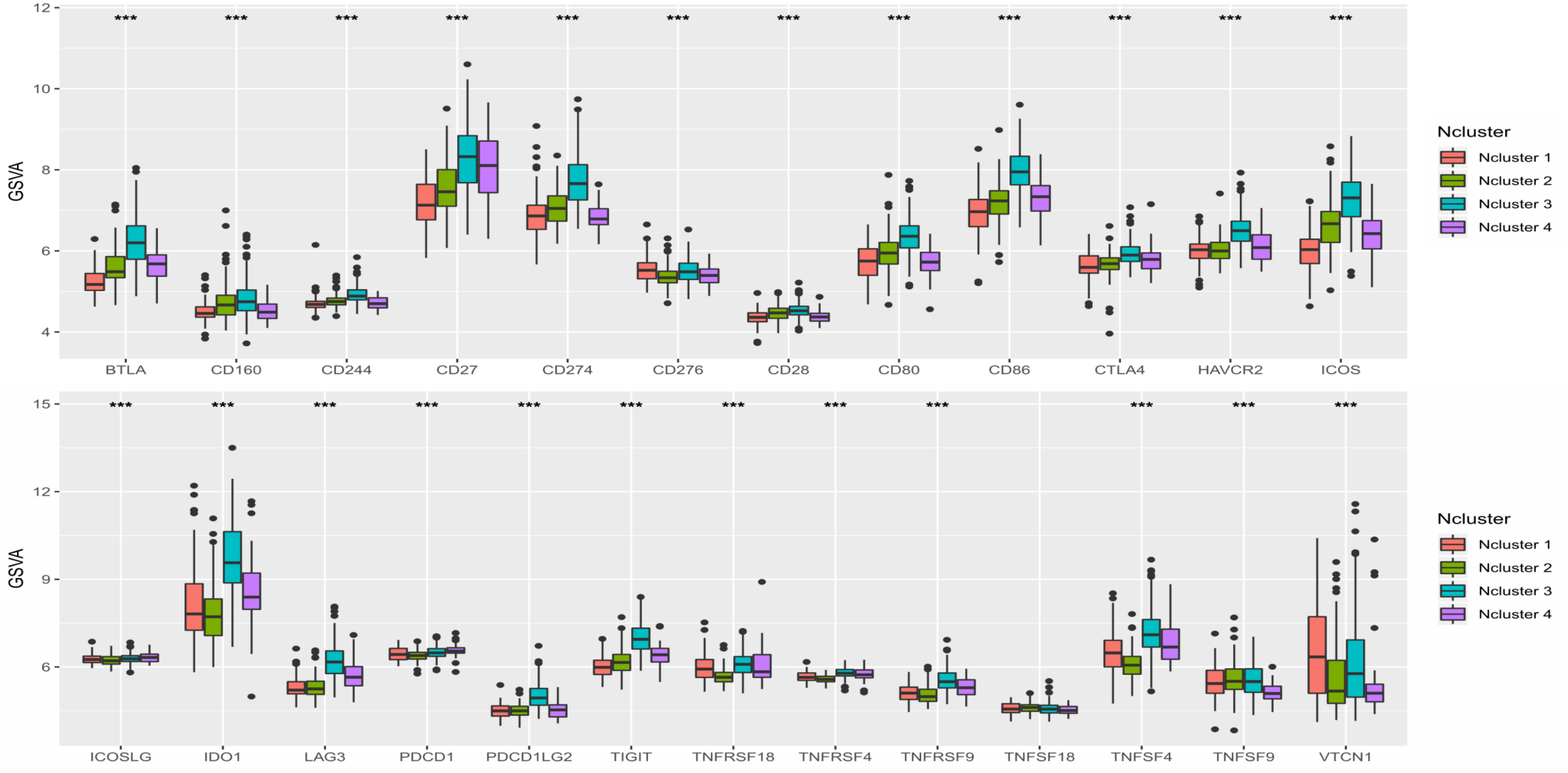

Figure S8. Boxplot of 25 immune checkpoint relevant molecules across different immune subtypes in the training group. The boxplots show the medians and dispersions of the samples of different immune subtypes for each immune checkpoint relevant molecules. P-values are the results of the one-way ANOVA for the different immune subtypes (NC1-NC4). *** $p<0.001$

Figure S9

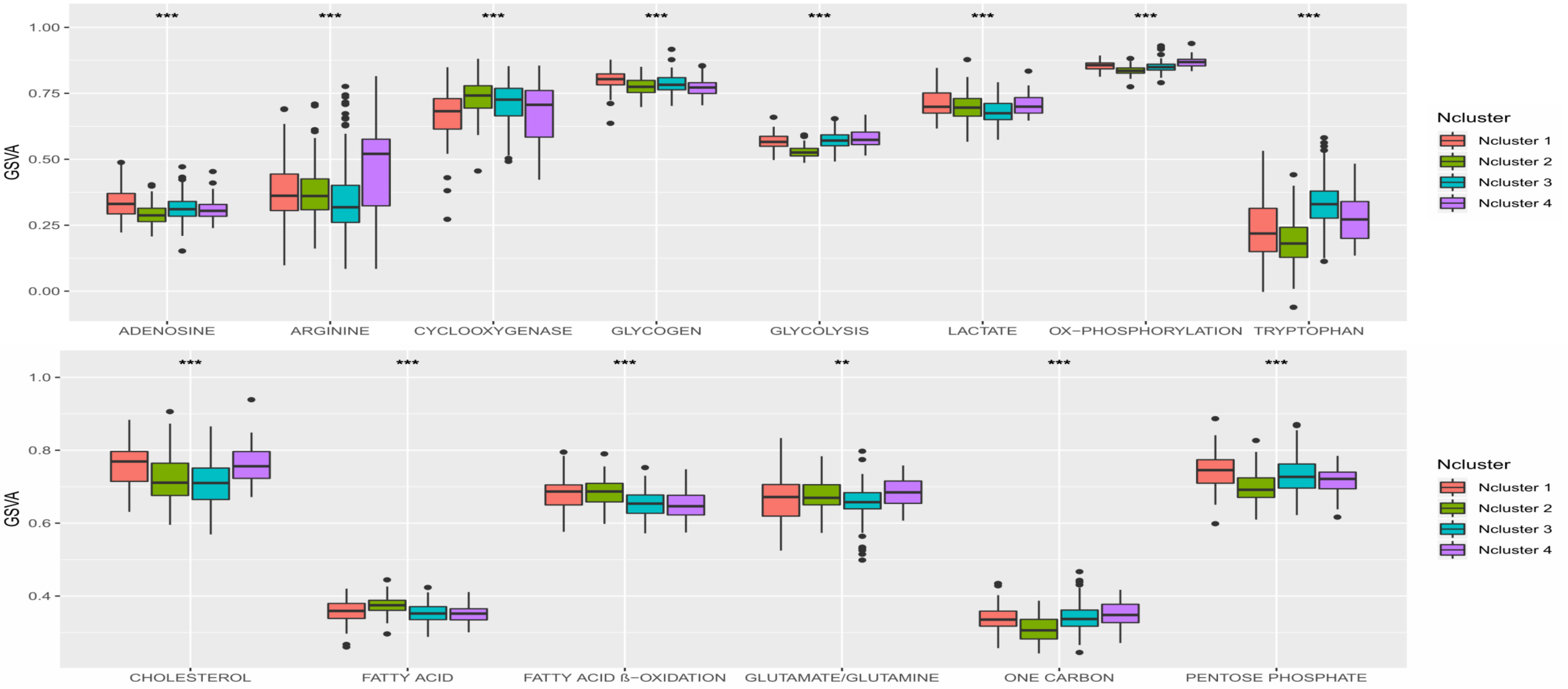

Figure S9. Boxplot of 14 metabolism pathways across different immune subtypes in the training group. The boxplots show the medians and dispersions of the samples of different immune subtypes for each metabolism pathways. P-values are the results of the one-way ANOVA for the different immune subtypes (NC1-NC4). *** p<0.001

Figure S10

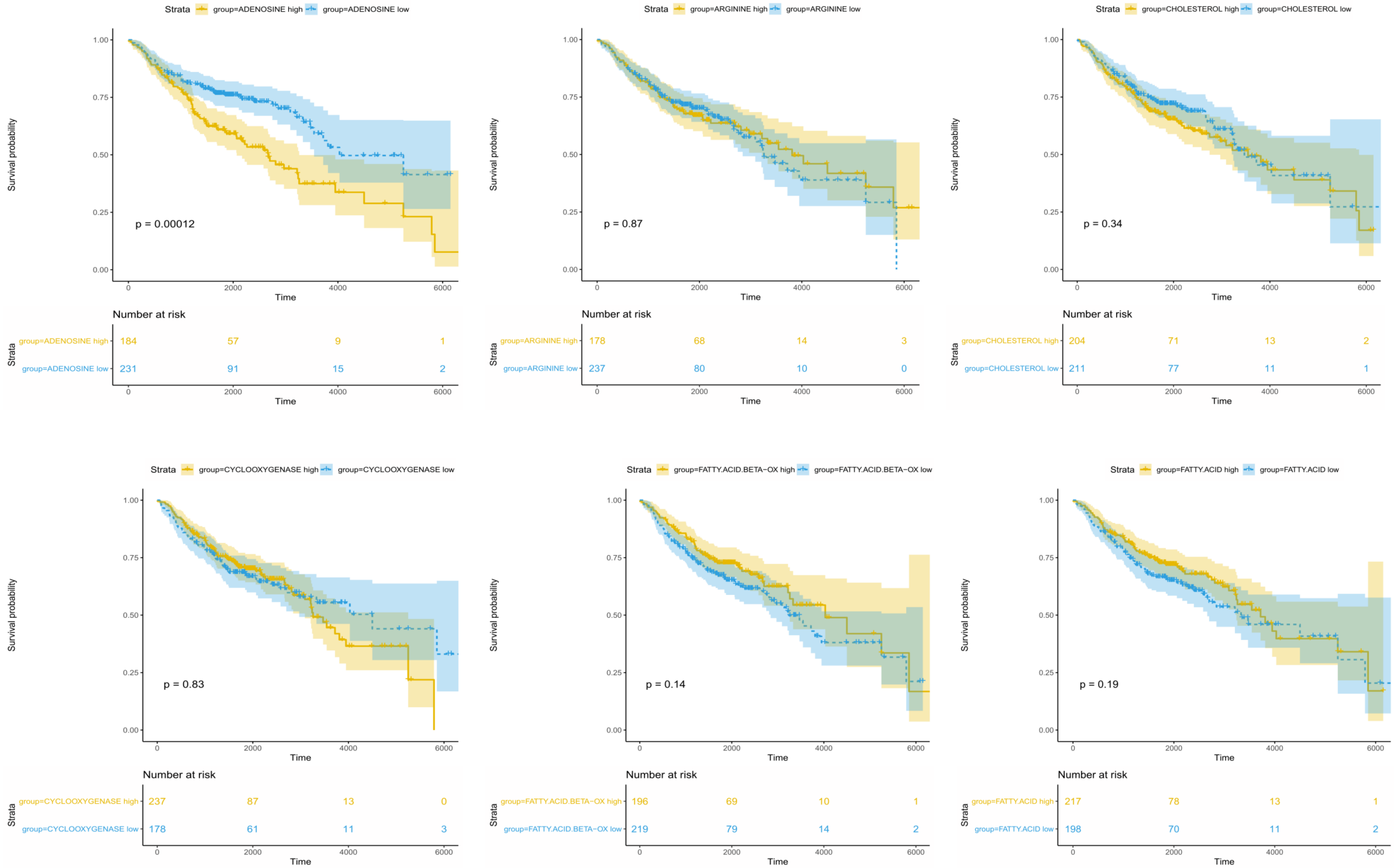

Strata group=ADENOSINE high group=ADENOSINE low
p = 0.00012
Number at risk
group=ADENOSINE high 184 57 9 1
group=ADENOSINE low 231 91 15 2
Strata group=ARGININE high group=ARGININE low
p = 0.87
Number at risk
group=ARGININE high 178 68 14 3
group=ARGININE low 237 80 10 0
Strata group=CHOLESTEROL high group=CHOLESTEROL low
p = 0.34
Number at risk
group=CHOLESTEROL high 204 71 13 2
group=CHOLESTEROL low 211 77 11 1
Strata group=CYCLOOXYGENASE high group=CYCLOOXYGENASE low
p = 0.83
Number at risk
group=CYCLOOXYGENASE high 237 87 13 0
group=CYCLOOXYGENASE low 178 61 11 3
Strata group=FATTY.ACID.BETA−OX high group=FATTY.ACID.BETA−OX low
p = 0.14
Number at risk
group=FATTY.ACID.BETA−OX high 196 69 10 1
group=FATTY.ACID.BETA−OX low 219 79 14 2
Strata group=FATTY.ACID high group=FATTY.ACID low
p = 0.19
Number at risk
group=FATTY.ACID high 217 78 13 1
group=FATTY.ACID low 198 70 11 2
Survival probability
Time
Strata

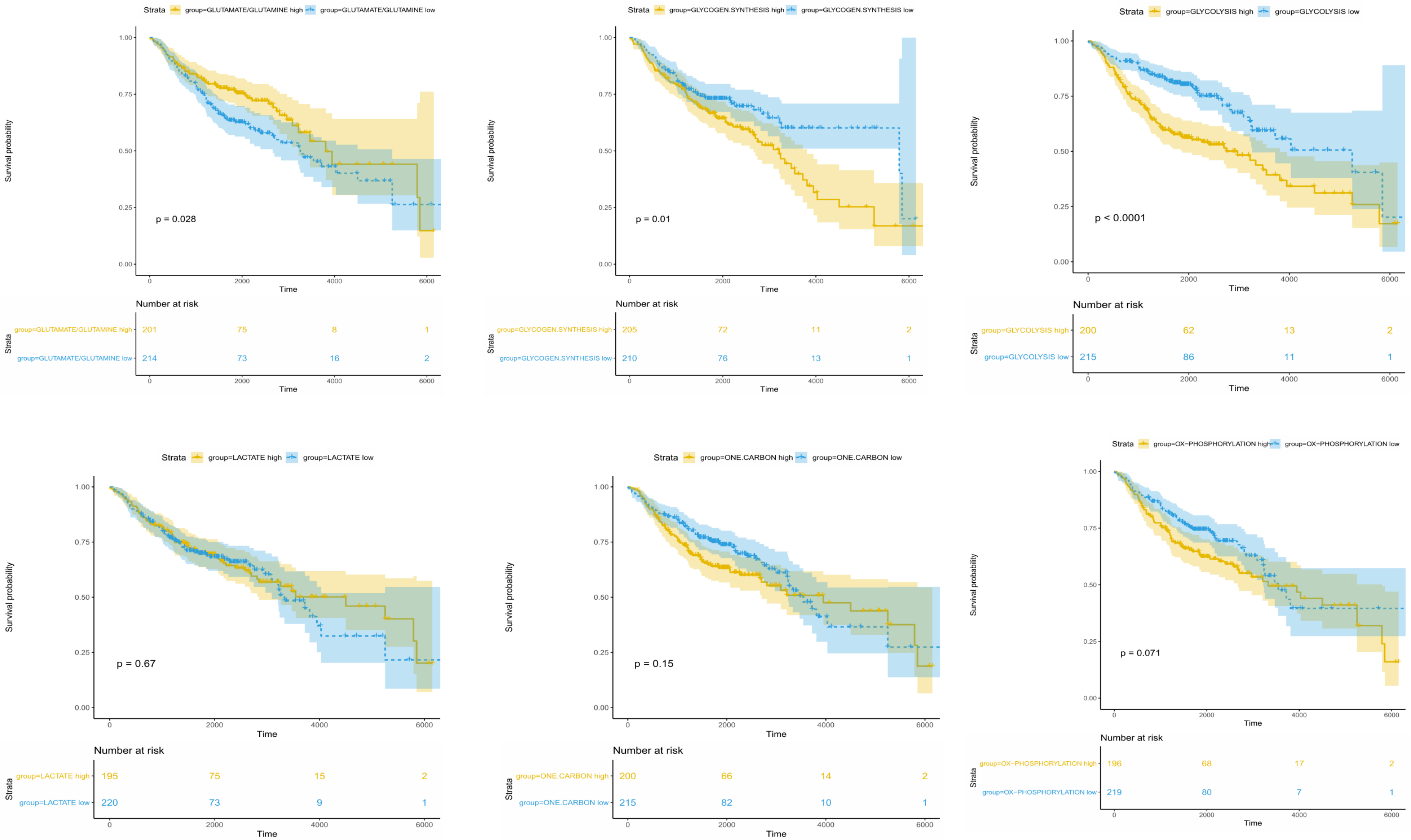
Strata group=GLUTAMATE/GLUTAMINE high group=GLUTAMATE/GLUTAMINE low
Survival probability
1.00
0.75
0.50
0.25
0.00
p = 0.028
0 2000 4000 6000
Time
Number at risk
group=GLUTAMATE/GLUTAMINE high 201 75 8 1
group=GLUTAMATE/GLUTAMINE low 214 73 16 2
Strata
Strata group=GLYCOGEN.SYNTHESIS high group=GLYCOGEN.SYNTHESIS low
p = 0.01
Number at risk
group=GLYCOGEN.SYNTHESIS high 205 72 11 2
group=GLYCOGEN.SYNTHESIS low 210 76 13 1
Strata group=GLYCOLYSIS high group=GLYCOLYSIS low
p < 0.0001
Number at risk
group=GLYCOLYSIS high 200 62 13 2
group=GLYCOLYSIS low 215 86 11 1
Strata group=LACTATE high group=LACTATE low
p = 0.67
Number at risk
group=LACTATE high 195 75 15 2
group=LACTATE low 220 73 9 1
Strata group=ONE.CARBON high group=ONE.CARBON low
p = 0.15
Number at risk
group=ONE.CARBON high 200 66 14 2
group=ONE.CARBON low 215 82 10 1
Strata group=OX−PHOSPHORYLATION high group=OX−PHOSPHORYLATION low
p = 0.071
Number at risk
group=OX−PHOSPHORYLATION high 196 68 17 2
group=OX−PHOSPHORYLATION low 219 80 7 1

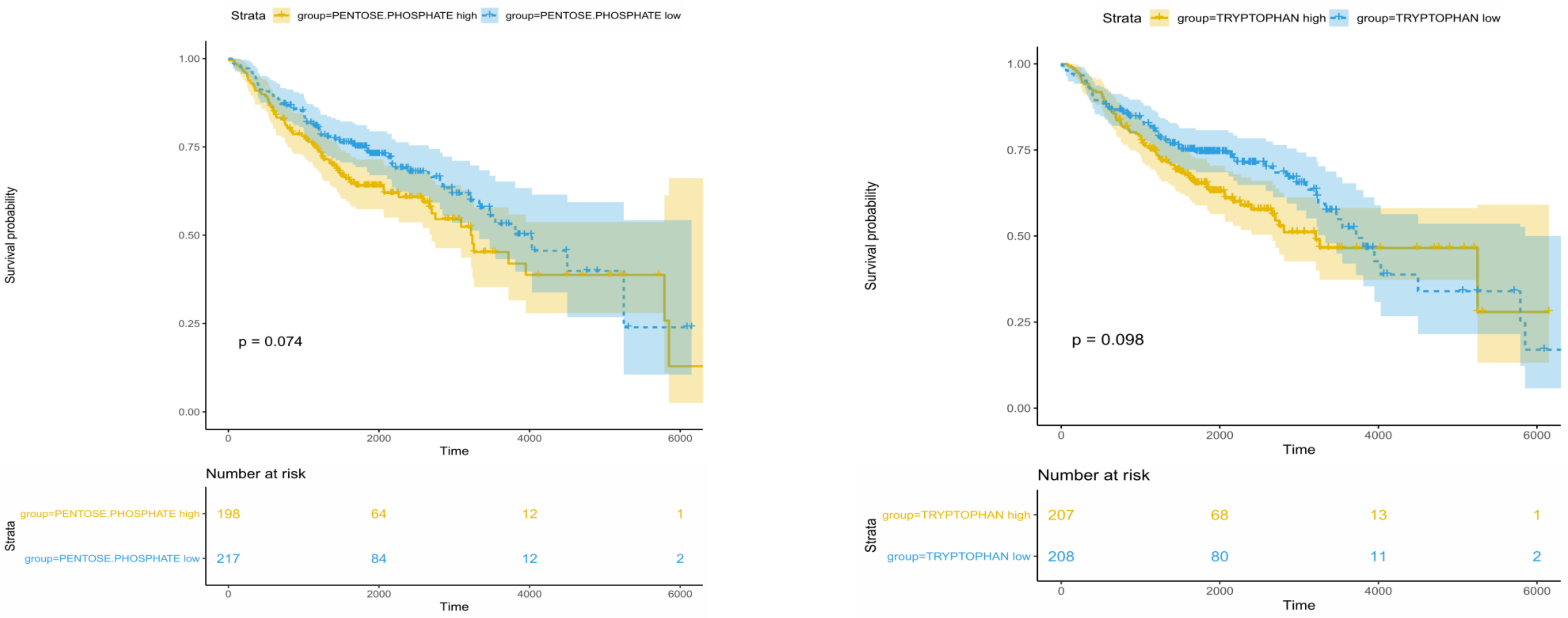

Figure S10. Kaplan-Meier plot of 14 metabolism pathways. After z-score standardization, values less than zero are classified as low group. These pathways include adenosine metabolic process, arginine biosynthetic, cyclooxygenase, fatty acid biosynthetic, lactate metabolic process, one carbon metabolic process, oxidative phosphorylation, cholesterol biosynthesis, glutamate and glutamine metabolism, glycogen synthesis, glycolysis, fatty acid beta oxidation, pentose phosphate pathway, and tryptophan catabolism.

Figure S11

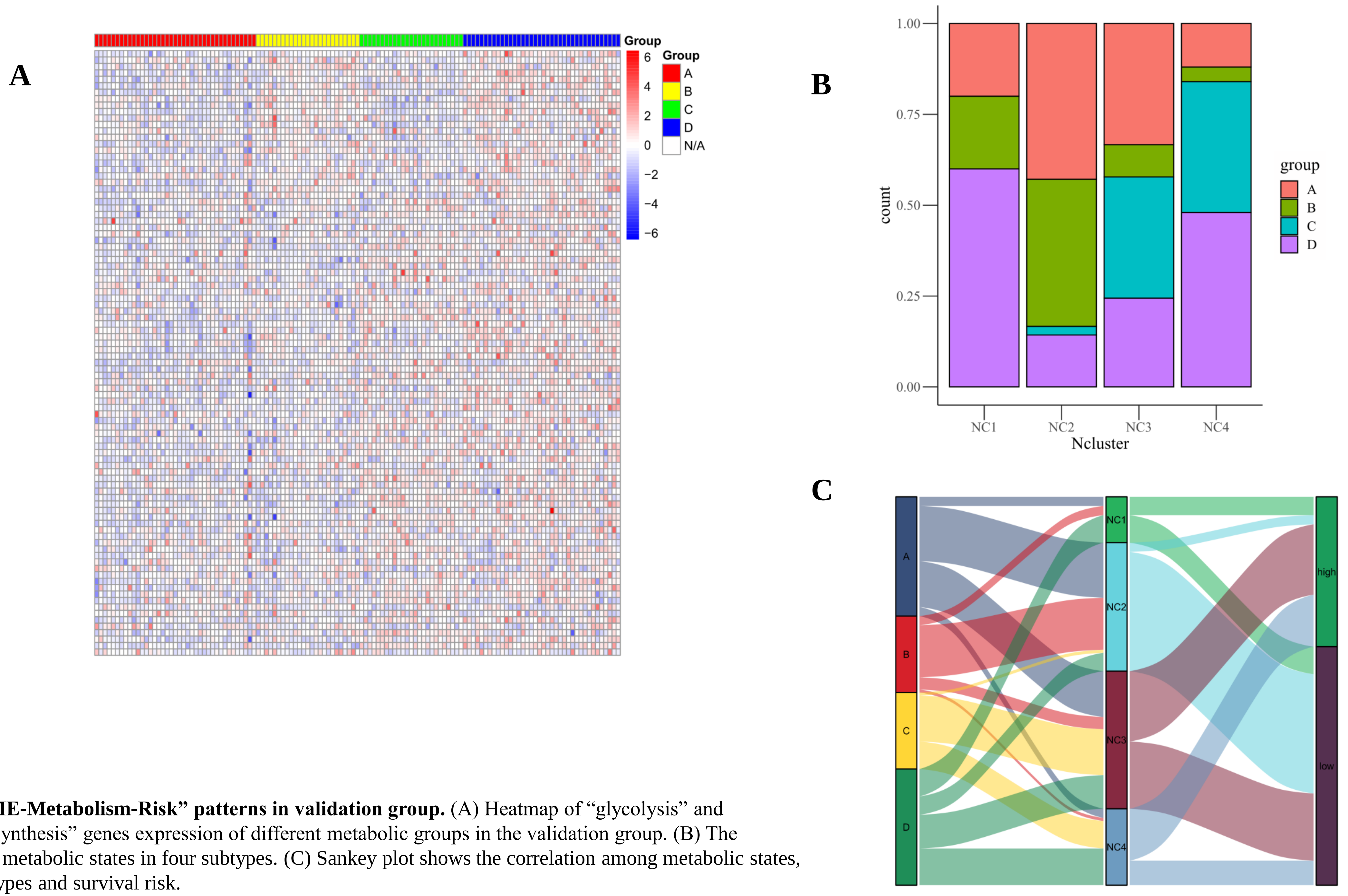

Fig S11. "TME-Metabolism-Risk" patterns in validation group. (A) Heatmap of "glycolysis" and "cholesterol synthesis" genes expression of different metabolic groups in the validation group. (B) The proportion of metabolic states in four subtypes. (C) Sankey plot shows the correlation among metabolic states, immune subtypes and survival risk.

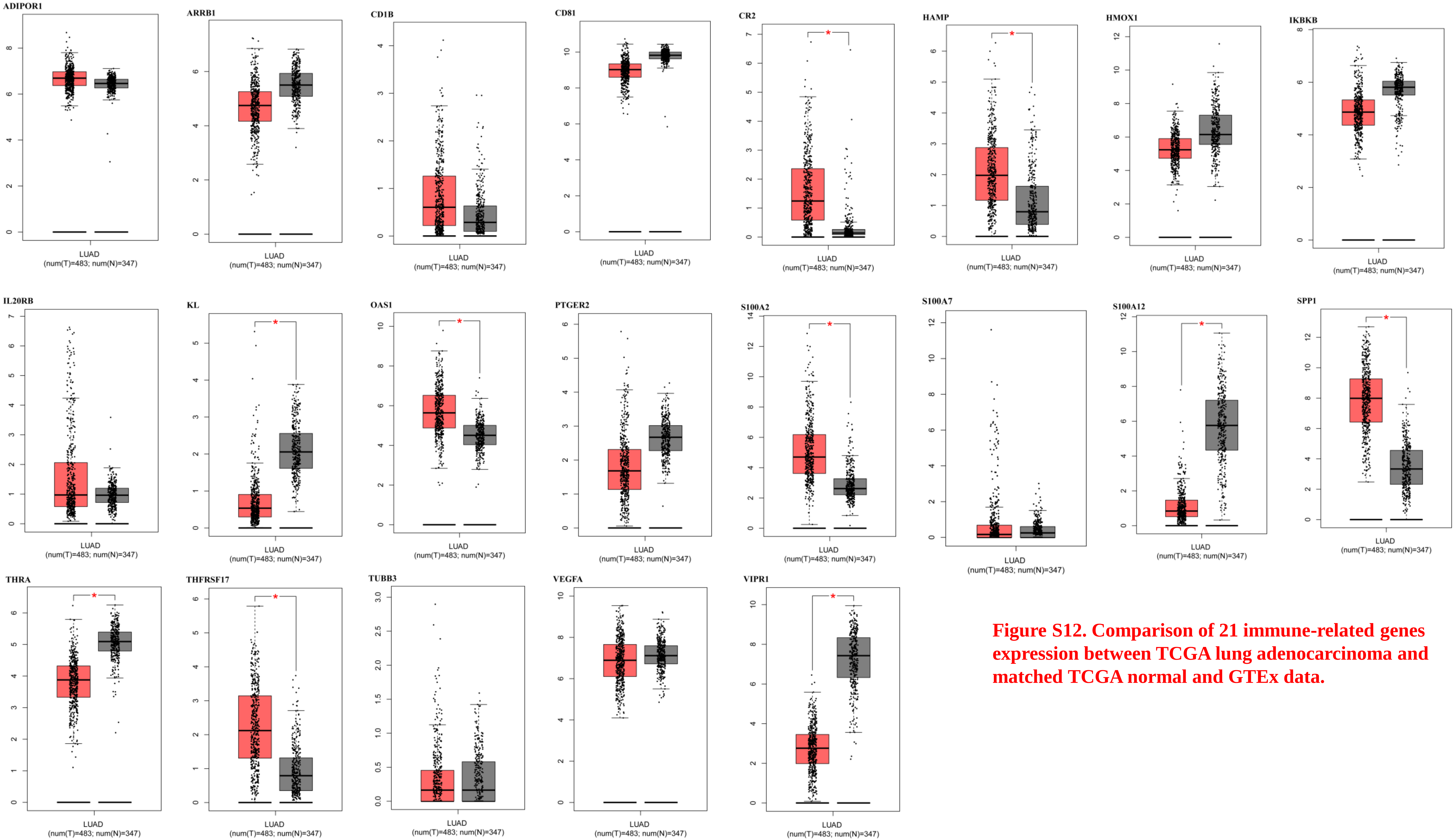

Figure S12. Comparison of 21 immune-related genes expression between TCGA lung adenocarcinoma and matched TCGA normal and GTEx data.
