# Supplementary material for: Comprehensive Profiling Reveals Distinct Microenvironment and Metabolism Characterization of Lung Adenocarcinoma
Source: Front Genet. 2021 May 28;12:619821. doi: 10.3389/fgene.2021.619821 (PMC8193848; doi:10.3389/fgene.2021.619821)
Supplement: Supplementary file 2 [file Data_Sheet_2.zip › Suppl Tables 1 - 6.DOCX]

## Table S1. Patients’ information in the training group.

| **Characteristic** |  | **No.cases** |
| --- | --- | --- |
| Age | Age>=60 | 240 |
|  | Age<60 | 175 |
| Gender | FEMALE | 199 |
|  | MALE | 216 |
| Stage | Stage I | 307 |
|  | Stage II | 80 |
|  | Stage III | 14 |
|  | Stage IV | 4 |
|  | Unknow | 10 |
| Relapse | No | 245 |
|  | Yes | 117 |
|  | Unknow | 53 |

## Table S2. Survival analyses of 24 TME cells in lung adenocarcinoma(Training group).

| Cell types | HR | CI_up | | CI_low | log_rank_p |
| --- | --- | --- | --- | --- | --- |
| **B.cells.memory** | 0.797 | 0.997 | 0.637 | | 0.047 |
| B.cells.naive | 0.921 | 1.149 | 0.738 | | 0.464 |
| Dendritic.cells.activated | 0.832 | 1.04 | 0.666 | | 0.107 |
| Dendritic.cells.resting | 0.934 | 1.166 | 0.748 | | 0.546 |
| Endothelial cells | 0.831 | 1.04 | 0.665 | | 0.106 |
| Eosinophils | 1.007 | 1.257 | 0.807 | | 0.95 |
| Fibroblasts | 1.245 | 1.556 | 0.996 | | 0.054 |
| Macrophages.M0 | 1.178 | 1.472 | 0.942 | | 0.151 |
| Macrophages.M1 | 1.108 | 1.383 | 0.887 | | 0.367 |
| Macrophages.M2 | 1.154 | 1.441 | 0.924 | | 0.208 |
| Mast.cells.activated | 1.053 | 1.314 | 0.844 | | 0.649 |
| Mast.cells.resting | 0.895 | 1.117 | 0.717 | | 0.327 |
| Monocytes | 0.971 | 1.212 | 0.778 | | 0.798 |
| **Neutrophils** | 1.433 | 1.796 | 1.143 | | 0.002 |
| NK.cells.activated | 1.026 | 1.281 | 0.822 | | 0.822 |
| NK.cells.resting | 0.982 | 1.226 | 0.787 | | 0.873 |
| Plasma.cells | 0.856 | 1.069 | 0.685 | | 0.17 |
| **T.cells.CD4.memory.activated** | 1.512 | 1.899 | 1.203 | | 0.001 |
| T.cells.CD4.memory.resting | 0.928 | 1.159 | 0.744 | | 0.51 |
| T.cells.CD4.naive | 0.826 | 1.033 | 0.66 | | 0.095 |
| T.cells.CD8 | 1.017 | 1.269 | 0.815 | | 0.882 |
| T.cells.follicular.helper | 0.871 | 1.087 | 0.697 | | 0.221 |
| T.cells.gamma.delta | 0.952 | 1.188 | 0.763 | | 0.661 |
| T.cells.regulatory..Tregs. | 1.15 | 1.437 | 0.92 | | 0.22 |
|  |  |  |  | |  |

## Table S3 Differentially expressed genes in NC3H compared to NC3L.

| **Regulation** | **Gene symbol** |
| --- | --- |
| Upregulated genes | AKR1B10, MMP1, SPP1, COL11A1, IL20RB, KRT6A, GPR87, SPOCK1, MMP12, CXCL5, TCN1, ANLN, CYP24A1, S100A9, GJB2 |
| Downregulated genes | SLC34A2, SELENBP1, C2orf40, FDCSP, C4BPA, SUSD2, AGR3, ZNF750, GPR133, AQP4, PEBP4, LOC286189, CPB2, MAMDC2, ZNF385B, LRRK2, NAPSA, PGC, CYP4B1, ADH1B, SCGB1A1, C16orf89, CYP2B7P, SFTPB, SCGB3A1, SFTPD, SFTA3, WIF1, SCGB3A2, SFTPC |

## Table S4. Patients’ information in the validation group.

| **Characteristic** |  | **No.cases** |
| --- | --- | --- |
| Age | Age>60 | 103 |
|  | Age<=60 | 24 |
| Gender | FEMALE | 62 |
|  | MALE | 65 |
| T | T1 | 43 |
|  | T2 | 82 |
|  | T3 | 2 |
| N | N0 | 94 |
|  | N1 | 33 |
| Stage | Stage I | 92 |
|  | Stage II | 35 |

## Table S5. Chi-square test in the training group.

| **Characteristics** | | **Subtype** | | | | | **χ^2^**  **(C1-C5)** | **P-value** | **χ^2^**  **(NC1-NC4)** | **P-value** |
| --- | --- | --- | --- | --- | --- | --- | --- | --- | --- | --- |
|  |  | C1 | C2 | C3 | C4 | C5 |  |  |  |  |
| **Gender** | Female | 43 | 68 | 8 | 64 | 16 | 15.495 | 0.0038 | 5.823 | 0.121 |
|  | Male | 38 | 57 | 31 | 67 | 23 |  |  |  |  |
| ***Status** | Alive | 42 | 100 | 23 | 75 | 20 | 24.127 | <0.001 | 24.089 | <0.001 |
|  | Dead | 39 | 25 | 16 | 56 | 19 |  |  |  |  |
| ***Stage** | Stage I | 55 | 113 | 21 | 95 | 23 | 34.140 | <0.001 | 26.337 | 0.002 |
|  | Stage II | 21 | 7 | 16 | 28 | 8 |  |  |  |  |
|  | Stage III | 3 | 3 | 1 | 5 | 2 |  |  |  |  |
|  | Stage IV | 1 | 1 | 0 | 1 | 1 |  |  |  |  |
| ***Relapse** | Yes | 32 | 17 | 19 | 38 | 11 | 27.694 | <0.001 | 23.269 | <0.001 |
|  | No | 40 | 96 | 17 | 74 | 18 |  |  |  |  |

## Table S6. Chi-square test in the validation group.

| **Characteristics** | | **Subtype** | | | | **χ^2^** | **P-value** |
| --- | --- | --- | --- | --- | --- | --- | --- |
|  |  | NC1 | NC2 | NC3 | NC4 |  |  |
| **Gender** | Female | 6 | 22 | 23 | 11 | 1.007 | 0.800 |
|  | Male | 9 | 20 | 22 | 14 |  |  |
| ***Status** | Alive | 9 | 33 | 25 | 9 | 12.389 | 0.006 |
|  | Dead | 6 | 9 | 20 | 16 |  |  |
| **Stage** | Stage I | 10 | 36 | 28 | 18 | 6.313 | 0.086 |
|  | Stage II | 5 | 6 | 17 | 7 |  |  |
